# Faradaic junction and isoenergetic charge transfer mechanism on semiconductor/semiconductor interfaces

**DOI:** 10.1038/s41467-021-26661-6

**Published:** 2021-11-04

**Authors:** Mingzhi Chen, Hongzheng Dong, Mengfan Xue, Chunsheng Yang, Pin Wang, Yanliang Yang, Heng Zhu, Congping Wu, Yingfang Yao, Wenjun Luo, Zhigang Zou

**Affiliations:** 1 Eco-materials and Renewable Energy Research Center (ERERC), National Laboratory of Solid State Microstructures, College of Engineering and Applied Sciences, Nanjing University, Nanjing, 210093 China; 2 Eco-materials and Renewable Energy Research Center (ERERC), Jiangsu Key Laboratory for Nano Technology, National Laboratory of Solid State Microstructures and Department of Physics, Nanjing University, Nanjing, 210093 China

**Keywords:** Photochemistry, Renewable energy

## Abstract

Energy band alignment theory has been widely used to understand interface charge transfer in semiconductor/semiconductor heterojunctions for solar conversion or storage, such as quantum-dot sensitized solar cells, perovskite solar cells and photo(electro)catalysis. However, abnormally high open-circuit voltage and charge separation efficiency in these applications cannot be explained by the classic theory. Here, we demonstrate a Faradaic junction theory with isoenergetic charge transfer at semiconductor/semiconductor interface. Such Faradaic junction involves coupled electron and ion transfer, which is substantively different from the classic band alignment theory only involving electron transfer. The Faradaic junction theory can be used to explain these abnormal results in previous studies. Moreover, the characteristic of zero energy loss of charge transfer in a Faradaic junction also can provide a possibility to design a solar conversion device with a large open-circuit voltage beyond the Shockley-Queisser limit by the band alignment theory.

## Abstract (web summary)

Energy band alignment theory is used to understand interface charge transfer in semiconductor/semiconductor junctions but many abnormal results cannot be well explained. Here, the authors demonstrate a Faradaic junction theory with isoenergetic charge transfer at semiconductor/semiconductor interfaces to explain these abnormal results.

## Introduction

Since an energy band-alignment theory on p–n junctions was first proposed in 1949^1^ (Supplementary Fig. 1), a Si solar cell was developed to convert solar energy to electricity^2^. According to the band-alignment theory, Shockley and Queisser proposed a method to calculate the efficiency limit of a p–n solar cell, which has been a guideline in this field^3^. Thereafter, several emerging low-cost semiconductor/semiconductor junctions, such as quantum-dot-sensitized solar cells^4–6^ and perovskite solar cells^7–10^, have been widely used for solar conversion. The band-alignment theory has also been used to calculate theoretical photovoltages of these emerging solar cells. For instance, a theoretical open-circuit voltage (V_OC_) in a quantum-dot-sensitized solar cell (Supplementary Fig. 2a) can be obtained from the difference between the conduction-band position of electron-transporting layer and redox potential^6^. Out of the expectation, some recent studies suggest that the experimental value of V_OC_ is higher than the theoretical value in a quantum-dot-sensitized solar cell. Moreover, the V_OC_ value changes with altering a quantum-dot sensitizer, but keeps constant with altering an electron-transporting material. These results are evidently inconsistent with the classic band-alignment theory^11,12^. Similar phenomena have also been observed in perovskite solar cells, in which the V_OC_ value is only dependent on the band position of a perovskite absorber and a hole-transporting layer, not on an electron-transporting layer (Supplementary Fig. 2b)^10^. Such abnormal V_OC_ of a quantum-dot-sensitized or perovskite solar cell does not follow the classic band-alignment theory, which is still unclear to date.

Moreover, semiconductor/semiconductor heterojunctions have been widely used to improve photocatalytic^13–17^ or photoelectrocatalytic^18–20^ performance. Two kinds of different interface charge-transfer mechanisms based on energy band alignment theory, type-II (Supplementary Fig. 2c) and direct Z-scheme heterojunction (Supplementary Fig. 2d), have been proposed to explain improved solar-conversion efficiency. In a type-II heterojunction, charge separation efficiency and photocurrent are enhanced significantly, which is mainly due to the built-in electric field at the interface. It is worthy to note that the charge separation in type-II heterojunction would decrease both reduction potential of electrons and oxidation potential of holes after interface charge transfer, and thereby leads to obvious photovoltage loss. However, some recent studies suggest that no energy loss of electrons is observed after interface charge transfer^17,21^. Therefore, a direct Z-scheme heterojunction mechanism was proposed to explain this phenomenon. For example, in a TiO_2_/CdS direct Z-scheme heterojunction, the electrons in the conduction band of TiO_2_ would recombine with the holes in the valence band of CdS, while the holes in TiO_2_ and the electrons in CdS can thus maintain their high oxidization and reduction potentials, respectively. However, no electrons could be photoexcited in TiO_2_ under visible-light irradiation, which would result in less enhancement in the performance of a TiO_2_/CdS heterojunction. Nevertheless, remarkable enhancement on the photocatalytic performance of TiO_2_/CdS Z-scheme heterojunction was observed under both the full-arc and visible-light illumination^22^. The inconsistent results aforementioned suggest that the classic band-alignment theory is not suitable to describe this abnormal high V_OC_ and charge-separation efficiency. Therefore, it is highly desirable to propose a theory to comprehend the interface charge transfer in these semiconductor/semiconductor junctions.

Herein, by using a TiO_2_/CdS junction as a model, we investigated interface charge-transfer mechanism by in situ XPS, (quasi) in situ UV–vis, (quasi) in situ Raman, and electrochemical-impedance spectroscopy (EIS). Accordingly, we find that a TiO_2_/CdS heterojunction is a Faradaic junction, which is totally different from classic type-II or Z-scheme heterojunction. When photogenerated electrons in the conduction band of CdS inject into TiO_2_ with H^+^ ions from electrolyte, an intrinsic Faradaic layer on the surface of TiO_2_ is reduced. The fast Faradaic reaction leads to much higher charge-separation efficiency in the TiO_2_/CdS Faradaic junction. The semiconductor/semiconductor Faradaic junction is also different from a semiconductor/farador junction in previous studies and few people consider a semiconductor as a farador with redox characteristic^23,24^. Very importantly, we also find the characteristic of isoenergetic interfacial charge transfer in the TiO_2_/CdS Faradaic junction, which can keep high reduction potential of the electrons in the semiconductor with higher conduction band. The Faradaic junction theory on semiconductor/semiconductor interface can be used to explain all these inconsistent results aforementioned in solar cells and photo(electro)catalysis fields. The theory is different from the classic band-alignment theory that was proposed over 70 years ago, which also provides a possibility to design a solar-conversion or storage device with a high V_OC_ even beyond the Shockley–Queisser limit.

## Results

### Characterization and photoelectrochemical performance of TiO_2_/CdS

**Fig. 1:**
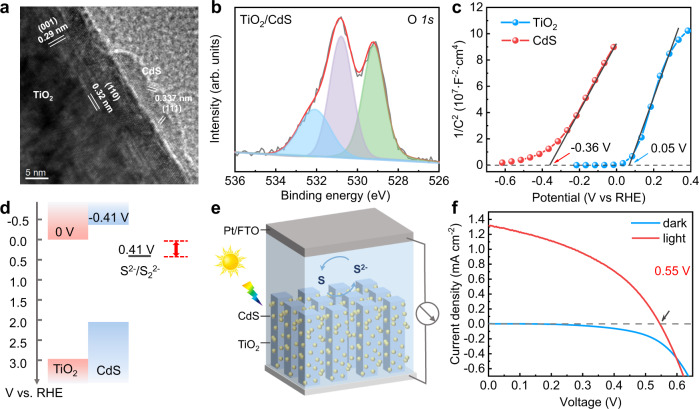
Characterization and V_OC_ of TiO_2_/CdS. **a** A TEM image of TiO_2_/CdS, (**b**) XPS spectra of O 1 s of TiO_2_/CdS, (**c**) Mott–Schottky plots of TiO_2_ and CdS in 1 M Na_2_S aqueous solution, (**d**) band positions of a TiO_2_/CdS heterojunction and redox potential of S^2−^/S, (**e**) a schematic diagram of a TiO_2_/CdS quantum-dot-sensitized solar cell, and (**f**) I–V curves of TiO_2_/CdS quantum-dot-sensitized solar cells under illumination and in the dark. Electrolyte: 0.5 M Na_2_S, 2 M S, and 0.2 M KCl in water/methanol (3:7 by volume). Light source: a Xe lamp with an AM 1.5 sunlight-simulator filter, light intensity: 100 mW/cm^2^.

A TiO_2_/CdS heterojunction was deposited on a FTO substrate by hydrothermal and successive ionic-layer adsorption and reaction method. Single TiO_2_ and CdS electrode were also prepared as reference samples. Supplementary Fig. 3 and Fig. 1a indicate the scanning electron microscope (SEM) and transmission electron microscope (TEM) images of a TiO_2_/CdS heterojunction. CdS particles are uniformly coated on TiO_2_ single-crystal nanorod arrays. The lattice spacing of 0.32 nm and 0.29 nm corresponds to the (110) and (001) of rutile TiO_2_, respectively. The observed 0.337-nm fringes on the surface of TiO_2_ nanorod correspond to the (111) of CdS. Figure 1b and Supplementary Fig. 4 indicate the X-ray photoelectron spectroscopy (XPS) patterns of TiO_2_/CdS. The binding energies of Ti^4+^, Cd^2+^, and S^2−^ peaks are observed on the surface of the sample, respectively. Similar to previous studies, the binding energies (529.2 eV, 530.9 eV, and 532.1 eV) of O 1 s are observed and assigned to the lattice O^2−^, lattice OH^−^, and adsorbed H_2_O molecules, which suggests that there is indeed a TiO_2−x_(OH)_2x_ layer on the surface of TiO_2_^25^.

In order to investigate the relationship between the V_OC_ of a solar cell and band alignment of different materials, the flat band potentials of TiO_2_ and CdS were measured by Mott–Schottky (M–S) method and the results are 0.05 V_RHE_ and −0.36 V_RHE_ (Fig. 1c). Generally, the conduction-band position is about 0.05 V higher than the flat-band potential^26^. Therefore, the conduction-band positions of TiO_2_ and CdS are 0 V_RHE_ and −0.41 V_RHE_, respectively, which are in good agreement with previous reports^27,28^. Similar band positions of TiO_2_ and CdS are also obtained by ultraviolet photoelectron spectroscopy and UV–visible absorption spectroscopy methods (Supplementary Fig. 5 and Table 1). Moreover, the redox potential of S^2−^/S was also measured as 0.41 V_RHE_ on a Pt/FTO electrode by cyclic voltammetry (CV) method (Supplementary Fig. 6). According to the results, the band positions of CdS, TiO_2_, and redox potential of S^2−^/S are plotted in Fig. 1d. From a classic band-alignment theory, a maximum theoretical V_OC_ of a CdS quantum-dot-sensitized solar cell is 0.41 V. In order to investigate the experimental V_OC_, a solar cell was assembled with a TiO_2_/CdS heterojunction as a photoanode, a Pt/FTO as a counter electrode, and polysulfide solution as electrolyte (Fig. 1e). The I–V curves of the solar cell were measured under illumination and in the dark, as shown in Fig. 1f. A V_OC_ of 0.55 V is obtained from the I–V curve, which is further confirmed by open-circuit potential method (Supplementary Fig. 7). The experimental V_OC_ is close to previously reported values^29^ and is higher than the theoretical value of 0.41 V. The V_OC_ of a quantum-dot-sensitized solar cell cannot be explained by classic band-alignment theory.

### Experimental evidence on a Faradaic reaction at TiO_2_/CdS interface

**Fig. 2:**
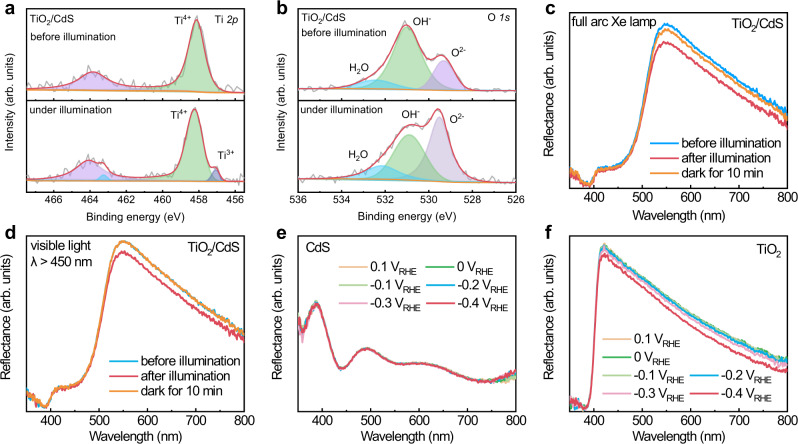
Charge-transfer process at TiO_2_/CdS interface. In situ XPS spectra of Ti 2p (**a**) and O 1 s (**b**) in TiO_2_/CdS in the dark and under full arc Xe lamp illumination, quasi in situ UV–vis reflectance spectra of TiO_2_/CdS in 1 M Na_2_S aqueous solution before and after illumination under full-arc Xe lamp (**c**) and visible light of *λ* > 450 nm (**d**), and in situ UV–vis reflectance spectra of CdS (**e**) and TiO_2_ (**f**) in 1 M Na_2_S aqueous solution at different potentials in the dark.

In order to clarify the reasons for abnormally high V_OC_, we investigated interface charge-transfer mechanism in TiO_2_/CdS by in situ XPS, (quasi) in situ UV–vis, (quasi) in situ Raman, and EIS method. In the dark, only Ti^4+^ is observed on the surface of TiO_2_/CdS, while some Ti^4+^ is reduced into Ti^3+^ under illumination (Fig. 2a). The decrease of the ratio of lattice OH^−^/lattice O^2−^ under illumination comes from oxidation of OH^−^ by photogenerated holes in TiO_2_ (Fig. 2b). Moreover, the binding energies of Cd 3d and S 2p do not change under illumination (Supplementary Fig. 8), which suggests that a chemical reaction only happens on the surface of TiO_2_ rather than CdS. In order to further investigate the reaction on the surface of TiO_2_/CdS, quasi in situ UV–vis reflectance spectra of a TiO_2_/CdS heterojunction were measured under open-circuit condition before and after full-arc Xe lamp illumination and the results are shown in Fig. 2c. After illumination, TiO_2_/CdS indicate lower reflectance at the wavelength range of 520–800 nm_._ In contrast, reflectance spectra of a single CdS and TiO_2_ electrode do not change after illumination (Supplementary Fig. 9). Moreover, a PL peak of CdS at 690 nm^30^ is quenched by contacting with TiO_2_ (Supplementary Fig. 10), which suggests that interface charge transfer happens between CdS and TiO_2_. The lower reflectance after illumination possibly comes from charge-transfer-induced change of TiO_2_/CdS. The same change trend of reflectance spectra was also observed when the TiO_2_/CdS was illuminated under visible light (*λ* > 450 nm), though TiO_2_ was not excited (Fig. 2d). Therefore, the visible light can still lead to the change of TiO_2_/CdS. In order to identify the change of reflectance from TiO_2_ or CdS, we measured in situ reflectance spectra of a single CdS and TiO_2_ electrode at different potentials in the dark (Fig. 2e, f). The reflectance spectra of a CdS electrode do not change at the potential range from −0.4 V_RHE_ to 0.1 V_RHE_, while the reflectance spectra of a TiO_2_ electrode decrease at the potentials negative than −0.2 V_RHE_, which comes from a reduction reaction of an intrinsic Faradaic layer of TiO_2−x_(OH)_2x_ (Ti^+4^O_2−x_(OH)_2x_ +H^+^ + e^−^ ↔ Ti^+3^O_1−x_(OH)_2x+1_) on the surface of TiO_2_^31^. The electron concentration in the surface Faradaic layer of TiO_2_ increases remarkably after reduction and leads to plasma-resonance absorption of free electrons at long wavelength^32^. Therefore, the lower reflectance in TiO_2_/CdS after illumination suggests that a reduction Faradaic reaction happens on the surface of TiO_2_.

In order to make clear why the Faradaic reaction happens only on the surface of TiO_2_, not CdS, CV and galvanostatic charge–discharge (GCD) curves of TiO_2_ and CdS were measured and the results are shown in Supplementary Fig. 11. Obvious hysteresis loop of a Faradaic reaction is observed in the CV curve of TiO_2_ but not in CdS (Supplementary Fig. 11a). The Faradaic potential window of TiO_2−x_(OH)_2x_ is further determined at the potential range from −0.4 V_RHE_ to 0.1 V_RHE_ by GCD method, while negligible Faradaic charge and discharge process is observed on CdS (Supplementary Fig. 11b). The results are in good agreement with previous study^31^. Therefore, a Faradaic reaction happens more easily on TiO_2_ than on CdS at the same reduction potential.

**Fig. 3:**
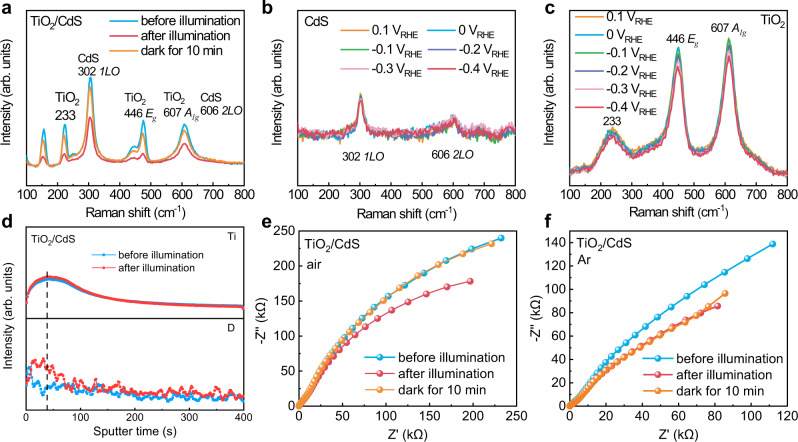
Interface change and ion-depth profiles in TiO_2_/CdS under illumination. **a** Quasi in situ Raman spectra of TiO_2_/CdS before and after illumination under full-arc Xe lamp, in situ Raman spectra of CdS (**b**) and TiO_2_ (**c**) in 1 M Na_2_S aqueous solution at different potentials in the dark, (**d**) secondary ion intensities of Ti and D ions in a TiO_2_/CdS junction in the electrolyte (1 M Na_2_S in D_2_O solution) before and after illumination for 10 min, electrochemical-impedance spectroscopy @0.4 V_RHE_ of TiO_2_/CdS in 1 M Na_2_S aqueous solution with air (**e**) and with Ar (**f**) before and after illumination under full-arc Xe lamp.

Quasi in situ Raman spectroscopy was also used to investigate interface changes^33^ in a TiO_2_/CdS heterojunction under illumination and the results are shown in Fig. 3a. The Raman peak intensity of TiO_2_/CdS decreases after illumination, which comes from the formation of amorphous Ti^+3^O_1−x_(OH)_2x+1_ on the surface of TiO_2_^34^. In contrast, the Raman peak intensities of single CdS and TiO_2_ do not change after illumination, which further confirm the interface charge transfer in TiO_2_/CdS (Supplementary Fig. 12). Similar to in situ UV–vis characterization in Fig. 2b and c, in situ Raman spectra of CdS and TiO_2_ were also measured at different potentials in the dark. The Raman peak intensity of CdS changes slightly (Fig. 3b), but the Raman peak intensity of TiO_2_ decreases with decreasing potentials (Fig. 3c). Therefore, the Raman spectra also suggest that charge transfer induced surface changes not on CdS but on TiO_2_ under illumination. Moreover, no obvious XRD change is observed in TiO_2_/CdS before and after illumination (Supplementary Fig. 13), which further confirms that the Faradaic reaction only happens on the surface of TiO_2_, not the bulk. In order to investigate the reaction process of H ions in the intrinsic Faradaic layer, isotope-labeling experiments by using D_2_O as a solvent were carried out and time-of-flight secondary-ion mass spectrometry (TOF-SIMS) was used to analyze depth profiles of D ions in the Faradaic junction before and after illumination. The results are shown in Fig. 3d. Ti ions are observed at the depth with sputtering time of 0–100 s and the distribution does not change before and after illumination. For D-ion depth profile, only possible adsorption of D ions on the surface of the sample before illumination, no D ions can be observed at the depth with sputtering time >10 s. After illumination, remarkably enhanced intensity of D ions is observed, and the distribution is in good agreement with the Ti ions, which suggest that D ions in the electrolyte insert into TiO_2_ under illumination. The concentration of H ions has significant effects on the efficiency of the TiO_2_/CdS heterojunction (Supplementary Fig. 14), which further supports the Faradaic junction mechanism at TiO_2_/CdS interface. Moreover, we find that the Faradaic reaction at TiO_2_/CdS interface not only happens in liquid phase, but also in gas-phase and solid-phase surroundings (Supplementary Figs. 15 and 16). Therefore, the Faradaic junction mechanism is universal to understand interface charge transfer in liquid photoelectrochemistry, gas photocatalysis, or solid-state solar cells.

Electrochemical-impedance spectroscopy (EIS) method was also used to investigate interface charge transfer in TiO_2_/CdS and the results are shown in Fig. 3e. In the dark, a TiO_2_/CdS heterojunction indicates similar EIS spectra with single TiO_2_, not CdS (Supplementary Fig. 17). Since CdS particles do not cover TiO_2_ nanorods completely and TiO_2_ contacts with electrolyte directly, the impedance spectra of TiO_2_/CdS mainly reflect the property of TiO_2_. The semicircle at low frequency of TiO_2_/CdS decreases obviously after illumination, which comes from the lower charge-transfer resistance in the TiO_2_/electrolyte interface^35^. In contrast, the impedance circle of single TiO_2_ and CdS does not change after the same illumination (Supplementary Fig. 18). Therefore, the decreased impedance semicircle of TiO_2_/CdS after illumination comes from the reduction reaction of an intrinsic Faradaic layer of TiO_2−x_(OH)_2x_ by photogenerated electrons from CdS_._ Moreover, since a Faradaic reaction is reversible, we also observed recovery process of UV–vis (Fig. 2c–d), Raman spectra (Fig. 3a) and EIS spectra (Fig. 3e) of TiO_2_/CdS when light was off and the sample was kept in electrolyte with air for 10 min. In contrast, no recovery process of EIS spectra was observed in the same electrolyte with Ar bubbling (Fig. 3f). The results suggest that the reduced intrinsic Faradaic layer on the surface of TiO_2_ can be reoxidized by dissolved oxygen in the electrolyte.

### A proposed mechanism

**Fig. 4:**
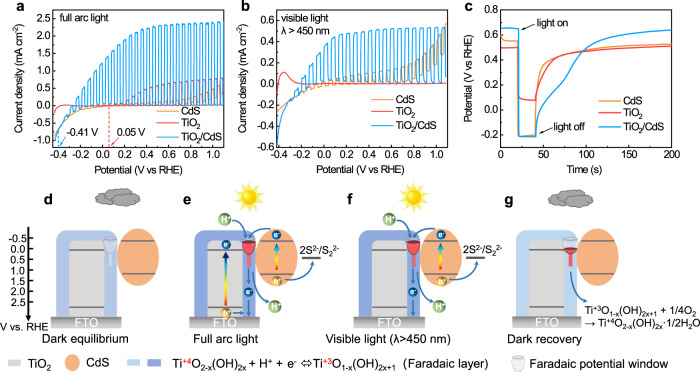
Characteristic and mechanism of isoenergetic charge transfer in a Faradaic junction. Linear-sweep voltammetry curves of TiO_2_, CdS and TiO_2_/CdS in 1 M Na_2_S aqueous solution under chopped full-arc Xe lamp illumination without (**a**) and with (**b**) a filter (wavelength >450 nm); (**c**) open-circuit potential of TiO_2_, CdS, and TiO_2_/CdS in 1 M Na_2_S aqueous solution with air in the dark and under illumination; (**d**–**g**) interface charge-transfer mechanisms in a TiO_2_/CdS heterojunction in the dark and under illumination.

Subsequently, we further study interface charge transfer characteristics of a TiO_2_/CdS Faradaic junction by linear-sweep voltammetry (LSV) and open-circuit potential (OCP) electrochemical methods. Figure 4a and b shows LSV curves of TiO_2_, CdS and TiO_2_/CdS in 1 M Na_2_S aqueous solution under full-arc and visible-light illumination, respectively. The photocurrent-onset potentials of TiO_2_, CdS, and TiO_2_/CdS are 0 V_RHE_, −0.41 V_RHE_ (Supplementary Fig. 19), and −0.41 V_RHE_, respectively, which are in good agreement with the conduction-band position in Fig. 1d. The onset potential of TiO_2_/CdS is the same to that of CdS, not TiO_2_, which suggests that the photogenerated electrons in CdS can be transferred to TiO_2_ without energy loss. Moreover, the TiO_2_/CdS indicates much higher stable photocurrent than those of TiO_2_ and CdS at different potentials, especially close to the onset potential of CdS (Supplementary Fig. 20). The results suggest that the TiO_2_/CdS heterojunction can not only keep the high reduction energy of photogenerated electrons of CdS, but also improve separation efficiency of photogenerated carriers simultaneously. An OCP method was also used to investigate interface charge-transfer process in the TiO_2_/CdS heterojunction (Fig. 4c). TiO_2_, CdS, and TiO_2_/CdS indicate different open-circuit potentials in the dark, which comes from chemical equilibrium between a sample surface and redox couples in the electrolyte. Under illumination, open-circuit potentials of the three samples are 0.07 V_RHE_, −0.2 V_RHE_, and −0.2 V_RHE_, respectively. The open-circuit potential of TiO_2_ under illumination is close to its onset potential. However, the open-circuit potential of CdS under illumination is 210 mV positive than its onset potential. The OCP is a potential with zero current, which indicates equilibrium potential of photooxidation for S^2−^ and dark reduction of S on a sample. There is much higher dark-reduction current of S than photooxidation for S^2−^ on CdS at the potential negative than −0.2 V_RHE_ (Supplementary Fig. 21a), which leads to the OCP of CdS pinning at −0.2 V_RHE_. In contrast, no obvious dark-reduction current of S is observed on TiO_2_ (Supplementary Fig. 21b). Therefore, the OCP of CdS under illumination is different from its onset potential, while the OCP of TiO_2_ under illumination is close to the onset potential. The TiO_2_/CdS indicates the same open-circuit potential under illumination to that of CdS, which further confirms that transferred electrons from CdS to TiO_2_ indicate the same energy to those in CdS. Moreover, when the light is off, slower decay process of OCP on TiO_2_ comes from reoxidization of the reduced-surface Faradaic layer by oxygen in the electrolyte (Supplementary Fig. 22). A TiO_2_/CdS heterojunction indicates the slowest decay process of the open-circuit potential among the three samples when the light is off. It is because of much negative potential of the photogenerated electrons in TiO_2_/CdS than that of TiO_2_, which leads to deeper reduction of the intrinsic Faradaic layer. Therefore, a TiO_2_/CdS heterojunction indicates an unusual isoenergetic interface charge transfer by a Faradaic reaction under illumination.

According to the above results and analysis, we propose an interface charge-transfer mechanism in a TiO_2_/CdS heterojunction in Fig. 4d–g. In the dark, there is an intrinsic Faradaic layer of Ti^+4^O_2−x_(OH)_2x_ at the interface of a TiO_2_/CdS heterojunction. The composition of the intrinsic Faradaic layer sensitively depends on potential and chemical equilibrium between a sample surface and redox couples in the electrolyte. Under illumination, photogenerated electrons in the conduction band of CdS transfer to TiO_2_, as well as H^+^ from the electrolyte, which reduce the intrinsic Faradaic layer (Ti^+4^O_2−x_(OH)_2x_) and raise its fermi level to the same position with the conduction-band bottom of CdS. Therefore, isoenergetic interfacial charge transfer is observed in a TiO_2_/CdS junction under illumination. The resistance of the reduced Faradaic layer (Ti^+3^O_1−x_(OH)_2x+1_) decreases remarkably and forms a short-circuit pathway for the electron transfer from the surface of TiO_2_ to a FTO substrate directly, not through the bulk of TiO_2_ (Fig. 4e), which is different from type-II heterojunction (Supplementary Fig. 23). Under full arc illumination, both of TiO_2_ and CdS are excited, the photogenerated electrons on TiO_2_ transfer to the FTO substrate through the bulk of TiO_2_, and the photogenerated holes diffuse to the surface of TiO_2_ and react with the reduced-surface Faradaic layer of Ti^+3^O_1−x_(OH)_2x+1_. Though a Faradaic reaction is observed at TiO_2_/CdS interface, the interface charge-transfer mechanism is also different from that in an indirect Z-scheme heterojunction with Au or redox shuttle as electron mediator (Supplementary Fig. 24)^36^. In a Faradaic junction, photogenerated electrons transfer from CdS to TiO_2_, however, reverse charge transfer direction in a Z-scheme junction. Under visible-light illumination (>450 nm), only CdS is excited (Fig. 4f), the reduced Faradaic layer of Ti^+3^O_1−x_(OH)_2x+1_ plays a role as a charge separator by a fast Faradaic reaction and an electron-transfer pathway, which can improve the photoelectrochemical performance of CdS. When the light is off, the reduced Faradaic layer can be oxidized by oxygen in the electrolyte and a reversible Faradaic reaction happens, which leads to the Fermi level recovering to the initial value before illumination (Fig. 4g). Therefore, the interface charge-transfer mechanism in a TiO_2_/CdS heterojunction is neither type-II heterojunction nor Z-scheme heterojunction, but a Faradaic junction, in which the characteristic of zero-energy loss and high charge-separation efficiency can be obtained at the same time by a fast and reversible Faradaic reaction.

In order to further investigate universality of the semiconductor/semiconductor Faradaic junction theory, we also prepared different oxide semiconductors (ZnO, Nb_2_O_5_, and Fe_2_O_3_) to replace TiO_2_ and constructed heterojunctions with CdS (Supplementary Figs. 26 and 27). Similar to TiO_2_/CdS, impedance semicircles of the ZnO/CdS, Nb_2_O_5_/CdS, and Fe_2_O_3_/CdS decrease after illumination, which also possibly come from reduction of intrinsic Faradaic layers of ZnO, Nb_2_O_5_ and Fe_2_O_3_ by photogenerated electrons from CdS (Supplementary Fig. 28). Moreover, ZnO/CdS and Nb_2_O_5_/CdS heterojunctions indicate close onset potentials to that of CdS. However, the onset potential of Fe_2_O_3_/CdS heterojunction indicates 0.56 V positive than that of CdS (Supplementary Figs. 29–30). These results suggest that isoenergetic charge transfer also happens at ZnO/CdS and Nb_2_O_5_/CdS interfaces, but not at Fe_2_O_3_/CdS interface. In order to explain this phenomenon, we measured the Faradaic potential windows of ZnO, Nb_2_O_5_, and Fe_2_O_3_ and the results are plotted in Supplementary Fig. 31. ZnO and Nb_2_O_5_ indicate similar Faradaic potential windows to that of TiO_2_, while the Faradaic potential window of Fe_2_O_3_ is 0.50 V positive than that of TiO_2_. When a potential negative than 0.03 V_RHE_ is applied on a Fe_2_O_3_ electrode, a corrosion reaction (Fe^+3^O_x_(OH)_3−2x_ + 3H^+^ + e^−^→ Fe^2+^ + (3−x)H_2_O) happens and Fe_2_O_3_ is dissolved into the electrolyte (Supplementary Fig. 32). Therefore, when the photogenerated electrons in the conduction band of CdS transfer to ZnO and Nb_2_O_5_, the intrinsic Faradaic layers can be charged and their fermi levels can be raised to the same position with the conduction-band bottom of CdS. The results can explain the V_OC_ dependent on a quantum-dot sensitizer but not on an electron-transporting material in quantum-dot-sensitized or perovskite solar cells^10–12^. However, when the photogenerated electrons in the conduction band of CdS transfer to Fe_2_O_3_, the photogenerated electrons will lead to dissolution of Fe_2_O_3_. Therefore, a prerequisite of isoenergetic charge transfer at semiconductor/semiconductor Faradaic interfaces is that the conduction-band position of an electron-donor semiconductor should overlap with a Faradaic potential window of an electron-acceptor semiconductor (Supplementary Fig. 33).

## Discussion

In summary, we propose a Faradaic junction mechanism on a TiO_2_/CdS heterojunction, which is substantively different from classic band-alignment theory on a semiconductor/semiconductor junction. The Faradaic junction mechanism is observed not only in liquid-phase, but also in gas-phase and solid-phase surroundings. Moreover, we find that a TiO_2_/CdS Faradaic junction indicates unusual isoenergetic interface charge transfer, which can be used to explain abnormal photovoltages or reduction/oxidation potential in quantum dot/perovskite solar cells and photo(electro)catalysis. We also propose a prerequisite of isoenergetic charge transfer in semiconductor/semiconductor Faradaic junctions. The Faradaic junction theory can offer a different perspective to design semiconductors with suitable Faradaic potential windows, but not conventional band positions, for high-performance solar energy conversion and storage.

## Methods

### Preparation of TiO_2_ nanorod films

TiO_2_ nanorod films were prepared on FTO substrates by hydrothermal method^25^. Briefly, 15 mL of deionized water and 15 mL of HCl were mixed and stirred for 10 min. Then, 0.45 mL of titanium butoxide was added. The solution was transferred to a Teflon-lined steel autoclave with inserting FTO substrates in it. The hydrothermal reaction was conducted at 150 ^o^C for 9 h. Finally, the deposited films were washed by deionized water and calcined at 450 ^o^C for 1 h in air.

### Preparation of CdS on TiO_2_ nanorod array by SILAR method

CdS QDs were grown on the TiO_2_ nanorod array by successive ionic-layer adsorption and reaction (SILAR) method^37,38^. Typically, the TiO_2_ nanorod array films were immersed in Cd(CH_3_COO)_2_ solution (0.1 M) for 2 min and rinsed with deionized water, and then immersed in Na_2_S solution (0.1 M) for another 2 min followed by another rinsing with deionized water. Such a SILAR cycle was repeated for 15 times to obtain TiO_2_/CdS samples. CdS on FTO substrates was also prepared by the same method as reference samples.

### Characterization of samples

X-ray diffraction (XRD smartlab 9 kW) was used to characterize the crystal structures of the samples. Scanning electron microscope (SEM Nano Nova S230) and transmission electron microscope (TEM Tecnai G2 F20) were used to investigate the morphologies of the samples. X-ray photoelectron spectroscopy (XPS) was performed on a K-Alpha instrument operating with an Al Kα X-ray source. The binding energy was calibrated by C1s peak at 284.6 eV. Photoluminescence spectra (PL) of the samples were collected by a Renishaw InVia Raman Micro-PL system with a 375-nm He–Cd laser. Time-of-flight secondary-ion mass spectrometry (TOF-SIMS 5 iontof, PHI NanoTOFII) was used to analyze depth profiles of ions in the Faradaic junction before and after illumination.

### In situ characterization of samples

In situ UV–vis reflection spectra (UV, Shimadzu UV-2550) and in situ Raman (Horiba T64000, excitation wavelength ~488 nm) in the dark were measured in a cell with a Pt wire and a saturated Ag/AgCl electrode as the counter and reference electrode, respectively. The electrolyte was 1 M Na_2_S aqueous solution (pH~13.3). An electrochemical analyzer (CHI 760e, Shanghai Chenhua) was used to control the potentials of the samples. Quasi in situ UV–vis reflection and Raman spectra were measured immediately after the photoelectrodes were illuminated by a Xe lamp in 1 M Na_2_S aqueous solution (pH~13.3) at an open-circuit potential.

In situ X-ray photoelectron spectroscopy (Thermofisher Escalab 250Xi) was performed under a full-arc Xe lamp. The prepared TiO_2_/CdS sample was kept in the dark before the test. The sample was immersed in 0.5 M Na_2_SO_3_ aqueous solution and dried in air. Before measurement, the sample was exposed to water vapor for 10 min, and then in situ XPS was measured on the sample in the dark and after 3-mins’ illumination.

### Photoelectrochemical measurement

The photoelectrochemical properties of the films were investigated in a three-electrode cell using an electrochemical analyzer (CHI 760e, Shanghai Chenhua) under a Xe lamp with an AM 1.5 sunlight-simulator filter illumination (light intensity: 100 mW/cm2). A Pt mesh and a saturated Ag/AgCl electrode were used as a counter electrode and a reference electrode, respectively. The electrolyte was 1 M Na_2_S aqueous solution (pH~13.3). A reversible hydrogen electrode (RHE) potential was obtained by the following formula: V_RHE_ = V_Ag/AgCl_ + 0.059*pH + 0.197. The quantum-dot-sensitized solar cell full device was assembled by using TiO_2_/CdS as a photoelectrode and a Pt-sputtered FTO as a counter electrode. The electrolyte was 0.5 M Na_2_S, 2 M S, and 0.2 M KCl in water/methanol (3:7 by volume)^39^. The I–V curves of solid-state solar cells were measured by Keithley 2450.

### Reporting summary

Further information on research design is available in the Nature Research Reporting Summary linked to this article.

## Supplementary information

Supplementary Information

Lasing Reporting Summary

## Data Availability

The data that support the findings of this study are available from the corresponding authors upon reasonable request.
