# Supplementary material for: Faradaic junction and isoenergetic charge transfer mechanism on semiconductor/semiconductor interfaces
Source: Nat Commun. 2021 Nov 4;12:6363. doi: 10.1038/s41467-021-26661-6 (PMC8569189; doi:10.1038/s41467-021-26661-6)
Supplement: Supplementary file 1 — Supplementary Information [file 41467_2021_26661_MOESM1_ESM.pdf]

Supplementary Information

Faradaic Junction and Isoenergetic Charge Transfer Mechanism on Semiconductor/Semiconductor Interfaces

Mingzhi Chen [1]†, Hongzheng Dong [1]†, Mengfan Xue [2]†, Chunsheng Yang [1], Pin Wang [2], Yanliang Yang [1], Heng Zhu [1], Congping Wu [2], Yingfang Yao [1], Wenjun Luo [1,*], Zhigang Zou [1, 2]

[1]Eco-materials and Renewable Energy Research Center (ERERC), National Laboratory of Solid State Microstructures, College of Engineering and Applied Sciences, Nanjing University, Nanjing 210093, China

[2]Eco-materials and Renewable Energy Research Center (ERERC), Jiangsu Key Laboratory for Nano Technology, National Laboratory of Solid State Microstructures and Department of Physics, Nanjing University, Nanjing 210093, China

†These authors contributed equally to this work.

*Email: wjluo@nju.edu.cn;

Methods

Preparation of CdS on TiO_2 nanorod array by chemical bath deposition (CBD) method

The TiO_2 samples were immersed in an aqueous solution containing 1.5 mM $CdSO_4$, 75 mM $SC(NH_2)_2$ and 2.3 M $NH_3 \cdot H_2O$. The deposition temperature was 60 ℃ and the deposition time was 20 min, respectively.

Preparation of a solid-state TiO_2/CdS solar cell

A precursor solution was prepared by dissolving 72.5 mg spiro-OMeTAD, 28.8 μL tert-butylpyridine (tBP) and 17.5 μL lithium bis(trifluoromethanesulfonyl) imide (Li-TFSI) into 13.8 mL chlorobenzene. And then the precursor solution was spin-coated on the TiO_2/CdS film at 4000 r. p. m. for 30 s. Moreover, in order to investigate the effects of Li^+ additive, we also prepared a precursor solution without Li-TFSI as a reference. Finally, an Au layer was deposited by thermal evaporation as a top contact for performance measurement.

Preparation of ZnO/CdS, Nb_2O_5/CdS, Fe_2O_3/CdS films

The ZnO thin films were prepared on FTO substrates by CBD method[1]. A seed layer was prepared on FTO substrates by spin coating with 1:2 molar ratio of $Zn(CH_3COO)_2$ and NaOH ethanol solution. And then 0.1 M $Zn(NO_3)_2 \cdot 6H_2O$ and 0.1 M HTMA were mixed in a beaker with 20 mL deionized water and stirred for 10 min. ZnO was deposited on the FTO substrates at 90 ºC for 2.5 h. Finally, the deposited films were washed by deionized water.

The Nb_2O_5 samples were prepared by annealing Nb sheet at 550 ºC for 30 min in air.

The Ti doped Fe_2O_3 nanorod films were prepared on FTO substrates by hydrothermal method[2]. Typically, 7.5 mmol $FeCl_3 \cdot 6H_2O$ and 50 μL $TiCl_3$ were mixed in 100 mL deionized water. Then 0.6 mL HCl was used to adjust the pH of the solution. The solution was transferred to a Teflon-lined steel autoclave with inserting FTO substrates in it. The hydrothermal reaction was conducted at 100 ºC for 4 h. Finally, the deposited films were washed by deionized water and calcined at 675 ºC for 1 h in air.

The ZnO/CdS, Nb_2O_5/CdS, Fe_2O_3/CdS heterojunctions were obtained by coating CdS

quantum-dots on ZnO, Nb_2O_5 and Fe_2O_3 films following the same SILAR method above.

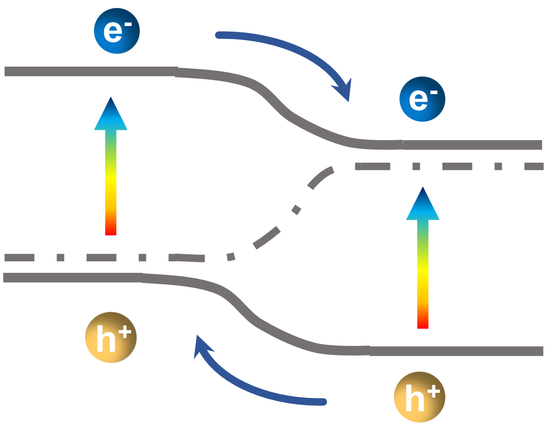

Supplementary Fig. 1| Classical energy band alignment theory for interface charge transfer in a p-n junction.

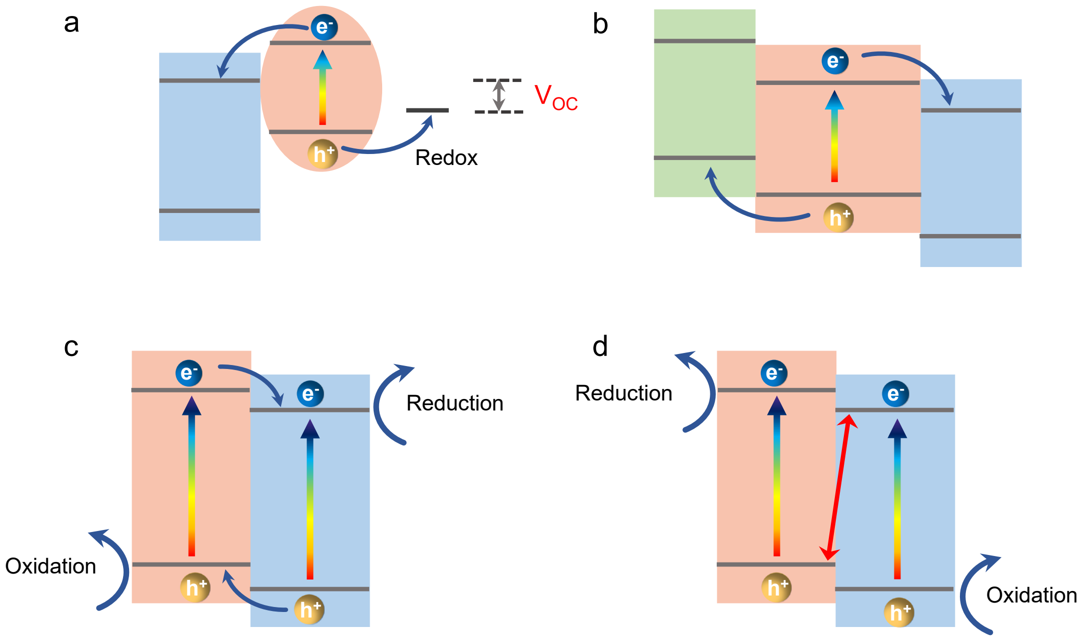

Supplementary Fig. 2| Classical energy band alignment theory for interface charge transfer in different emerging semiconductor/semiconductor junctions, quantum-dot sensitized solar cell (a), perovskite solar cell (b), type-II heterojunction (c) and direct Z-scheme heterojunction (d).

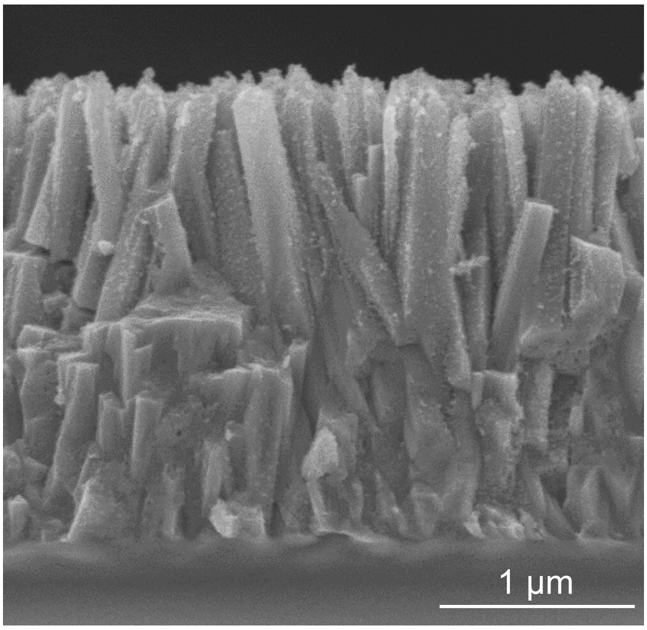

Supplementary Fig. 3| SEM images of TiO_2/CdS.

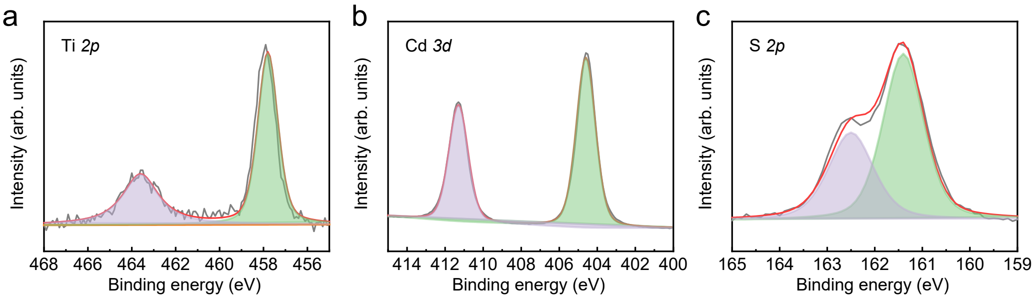

Supplementary Fig. 4| Schematic ex situ XPS spectra of a TiO_2/CdS heterojunction.

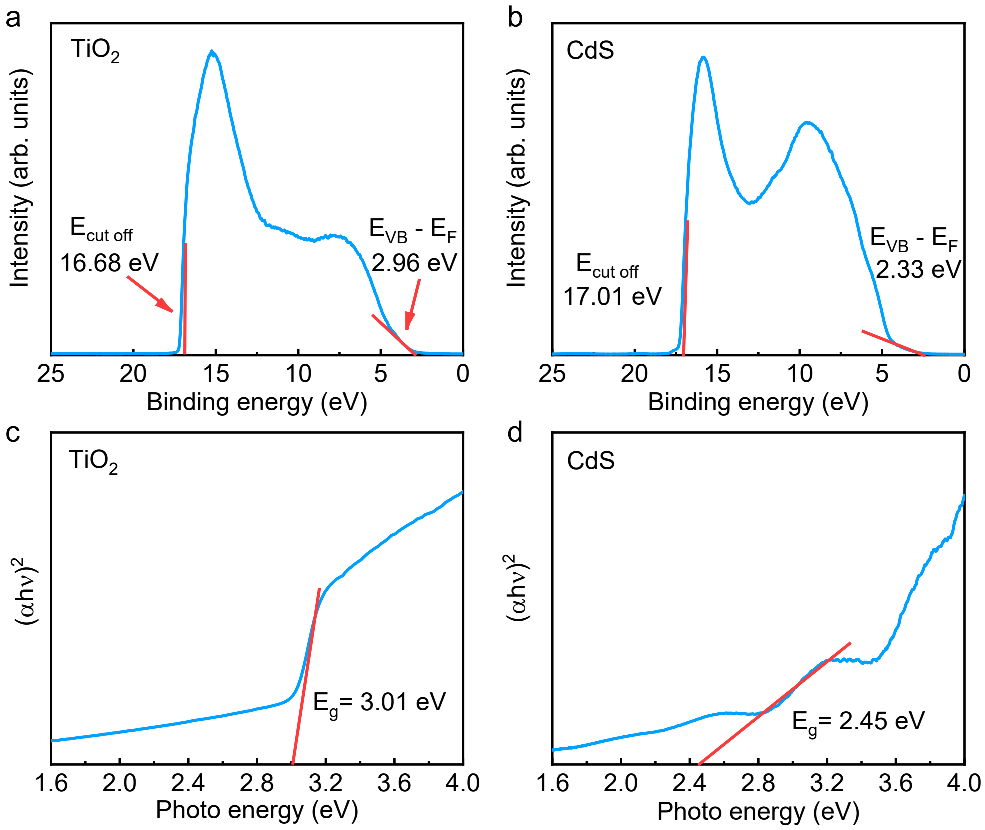

Supplementary Fig. 5| Ultraviolet photoelectron spectra of TiO_2 (a) and CdS (b); The Tauc plots of UV-visible absorption spectra of TiO_2 (c) and CdS (d); α, absorption coefficient; h, Planck's constant; ν, photo's frequency.[3,4]

	E_{CB} (vs. RHE)	E_{VB} (vs. RHE)	E_g (eV)
TiO_2	-0.03	2.98	3.01
CdS	-0.43	2.02	2.45

Supplementary Table 1| Band gaps and positions of TiO_2 and CdS determined by ultraviolet photoelectron spectroscopy and UV-visible absorption spectroscopy methods.

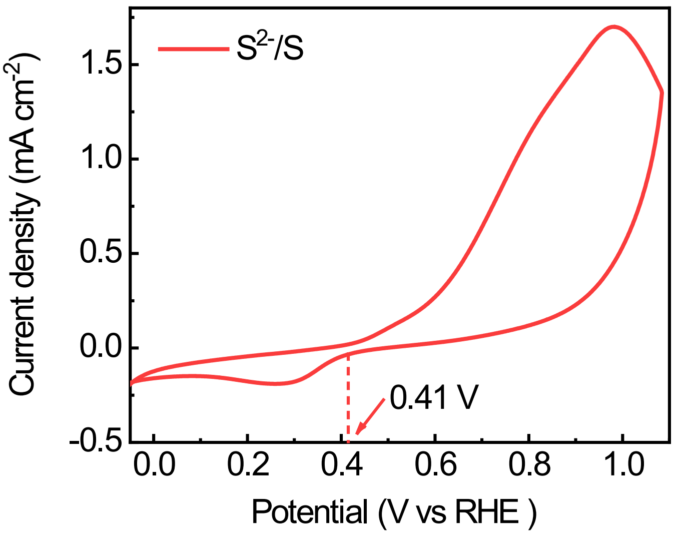

Supplementary Fig. 6| Cyclic voltammetry curve of S^{2-}/S on a Pt/FTO sample in 0.1 M Na_2S and 0.1 M S aqueous solution.

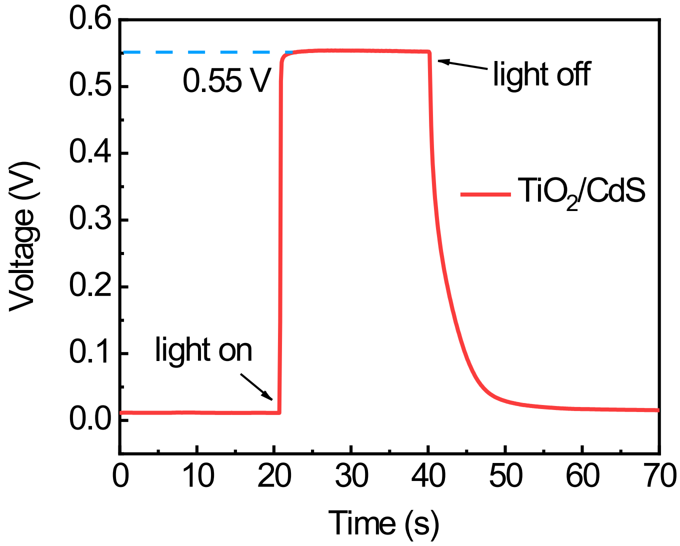

Supplementary Fig. 7| V_{OC} of TiO_2/CdS quantum-dot sensitized solar cells. Electrolyte: 0.5 M Na_2S, 2 M S and 0.2 M KCl in water/methanol (3:7 by volume). Light source: a Xe lamp with an AM 1.5 sunlight simulator filter, light intensity: 100 mW/cm^2.

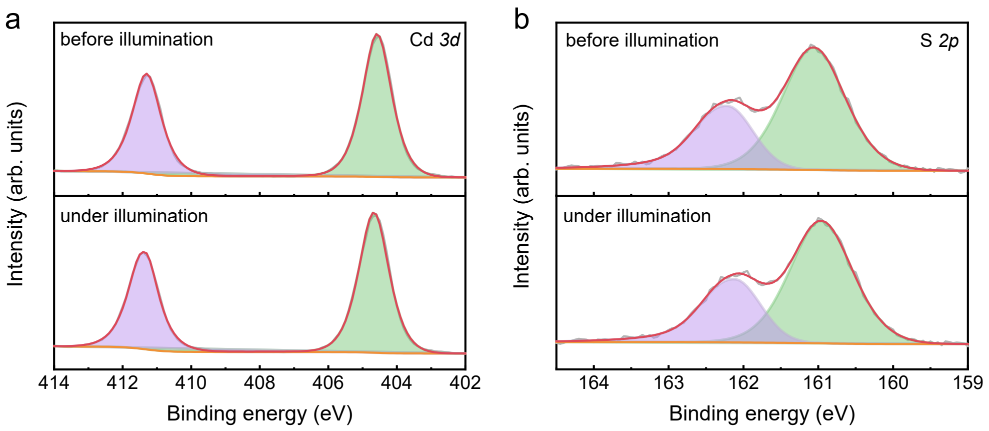

Supplementary Fig. 8| In situ XPS spectra of (a) Cd 3d and (b) S 2p in a TiO_2/CdS heterojunction in the dark and under illumination; Light source: a full arc Xe lamp.

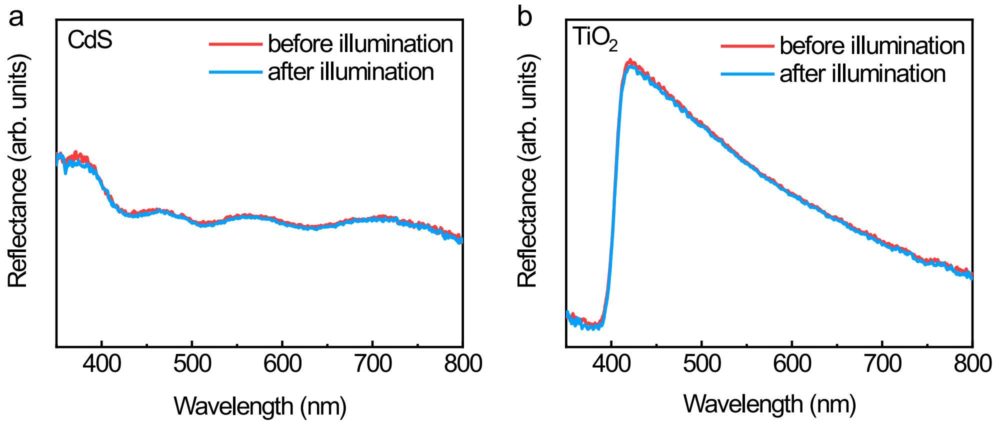

Supplementary Fig. 9| UV-vis spectra of CdS (a) and TiO_2 (b) in 1 M Na_2S aqueous solution before and after illumination; Light source: a full arc Xe lamp.

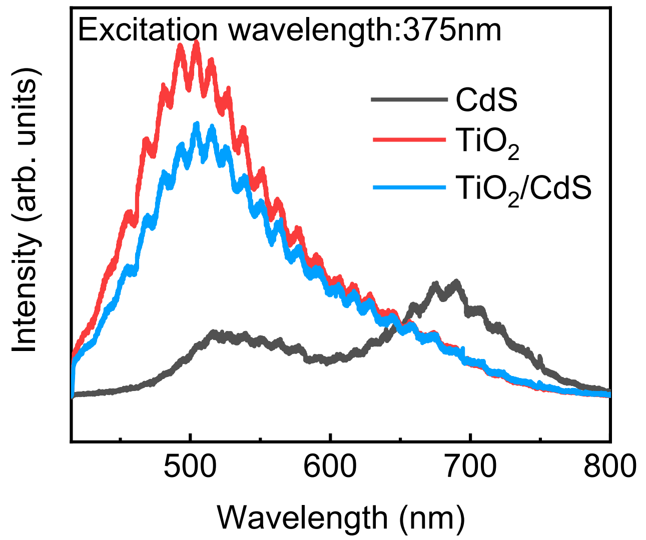

Supplementary Fig. 10| Photoluminescence spectra of CdS, TiO_2 and TiO_2/CdS, excitation wavelength: 375 nm.

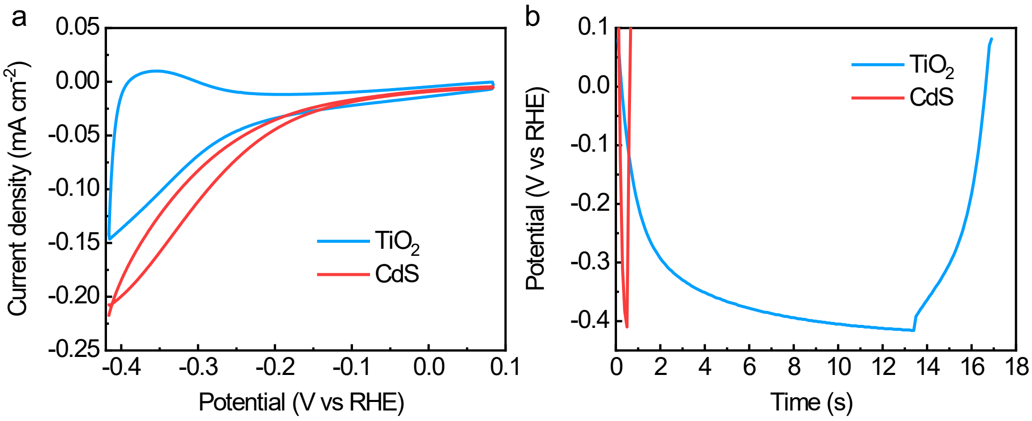

Supplementary Fig. 11| Cyclic voltammetry curves (a) and Galvanostatic charge-discharge curves (b) of TiO_2 and CdS in 1 M Na_2S aqueous solution in the dark.

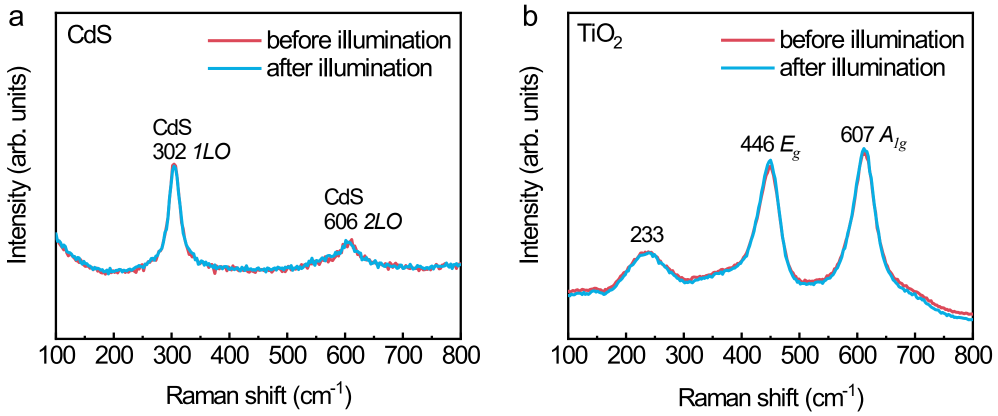

Supplementary Fig. 12| In situ Raman spectra of (a) CdS and (b) TiO_2 in 1 M Na_2S aqueous solution before and after illumination; Raman excitation wavelength: 488 nm; Light source: a full arc Xe lamp.

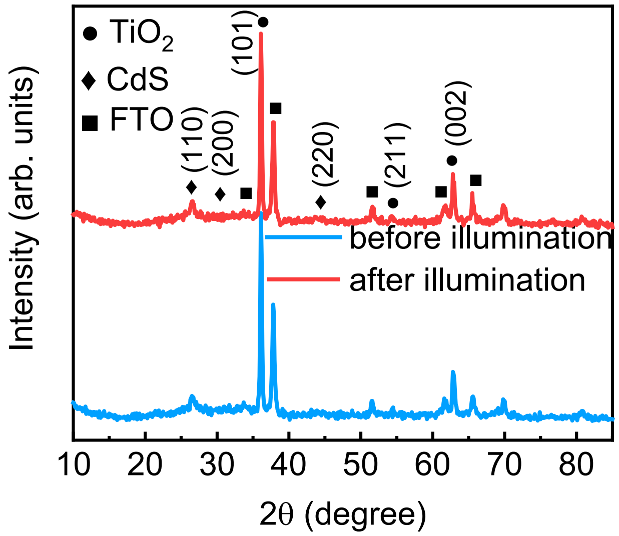

Supplementary Fig. 13| XRD of a TiO_2/CdS heterojunction before and after illumination in 1 M Na_2S aqueous solution. Light source: a full arc Xe lamp.

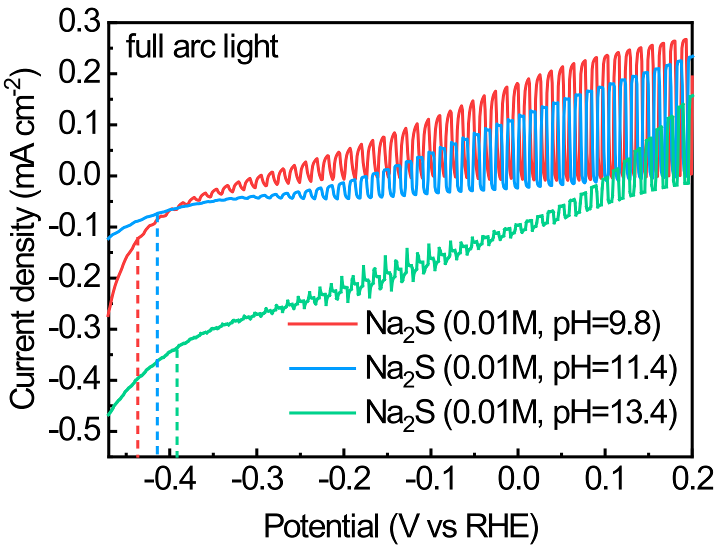

Supplementary Fig. 14| Linear sweep voltammetry curves of TiO_2/CdS in 0.01 M Na_2S aqueous solution with different pH values under chopped illumination. Light source: a Xe lamp with an AM 1.5 sunlight simulator filter, light intensity: 100 mW/cm^2.

Since protons participate in the reaction under illumination, we investigated the effects of pH values on the efficiency of the Faradaic junctions. The results are shown in Supplementary Fig. 14. As the concentration of H^+ increases, the pH value decreases, and the onset potential of TiO_2/CdS shifts negatively and the photocurrent increases obviously, which suggests that the pH values have significant effects on the efficiency of the Faradaic junctions. In the Faradaic reaction, $Ti^{+4}O_{2-x}(OH)_{2x} + H^+ + e^- \leftrightarrow Ti^{+3}O_{1-x}(OH)_{2x+1}$, the higher H^+ concentration, the faster the reaction rate.

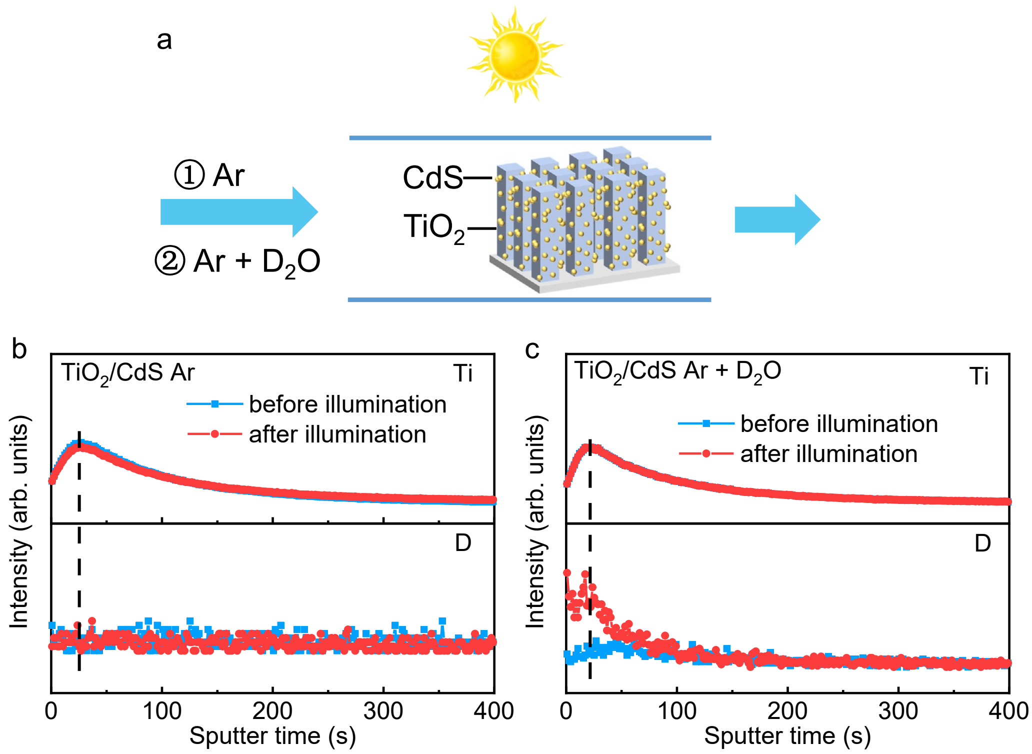

Supplementary Fig. 15| Schematic illustration of the experimental setup for isotope labeling experiments in photocatalysis conditions containing gas phase (a). Secondary ion intensity of D ions in TiO_2/CdS junction before and after illumination with dry Ar (b) or Ar/D_2O (c) as flow gas for 10 min. Light source: a Xe lamp with an AM 1.5 sunlight simulator filter, light intensity: 100 mW/cm^2.

In order to investigate whether the Faradaic junction mechanism is also suitable for gas phase photocatalysis, isotopic labeling experiments were also performed (Supplementary Fig. 15a) and the results are shown in Supplementary Fig. 15b-c. In dry Ar flow gas, no D ions are observed in the TiO_2/CdS heterojunction both before and after illumination. In contrast, D ions occur in the sample after illumination with Ar/D_2O as flow gas, which has similar distribution of the Ti ions. The inserted D ions come from water vapor not liquid water. These results suggest that the Faradaic reaction also happens in gas phase photocatalysis.

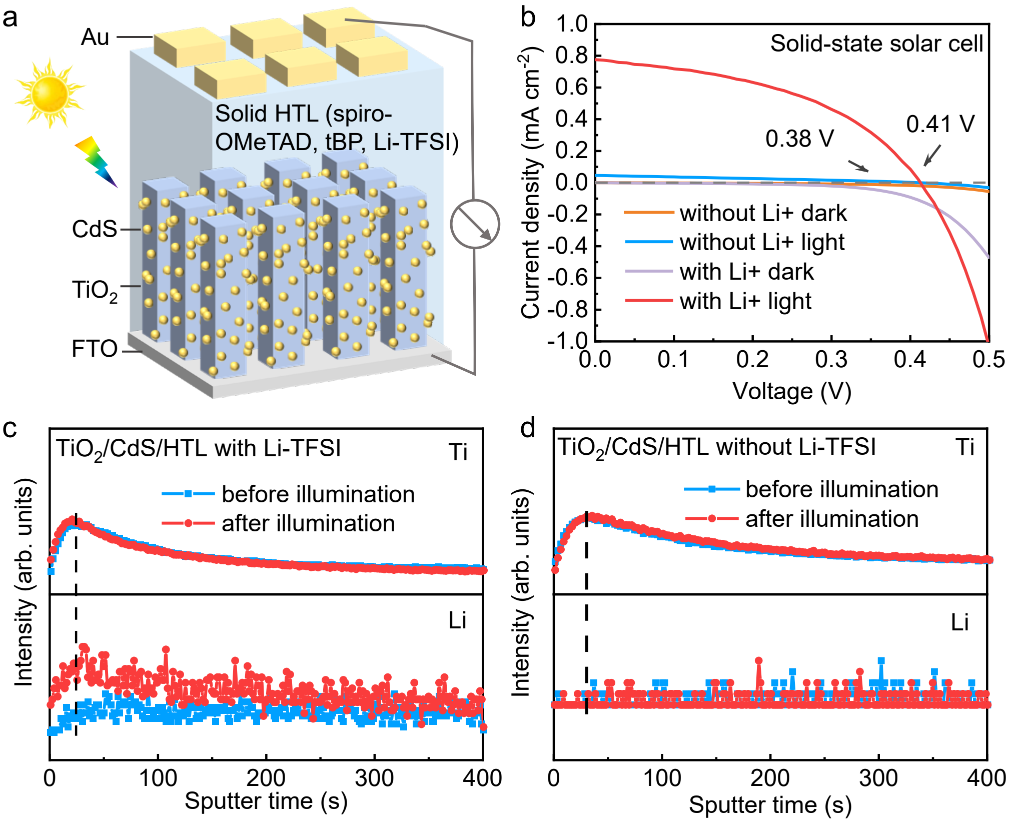

Supplementary Fig. 16| (a) A schematic diagram of a solid-state TiO_2/CdS quantum-dot sensitized solar cell; (b) I-V curves of solid-state TiO_2/CdS solar cells with and without Li-TFSI additive in the hole-transporting layer in the dark and under a Xe lamp illumination; Secondary ion intensity of Ti and Li in solid-state TiO_2/CdS solar cells with (c) or without (d) Li-TFSI before and after full arc Xe lamp illumination. Light source: a Xe lamp with an AM 1.5 sunlight simulator filter, light intensity: 100 mW/cm^2.

In order to investigate whether the Faradaic junction mechanism is also suitable for a solid-state solar cell, a solid-state TiO_2/CdS solar cell (Supplementary Fig. 16a) was also prepared following previous studies.[5] Spiro-OMeTAD was used as a solid hole-transporting layer (HTL) and tert-butylpyridine (tBP) and lithium bis(trifluoromethanesulfonyl)imide (Li-TFSI) as additives. The preparation details of the solid-state devices are shown in Methods. In order to investigate the effects of Li^+ additive, we also prepared a precursor solution without Li-TFSI as a reference. The I-V curves of the solar cells with and without Li-TFSI additive are shown in Supplementary Fig. 16b. The results suggest that the Li^+ ion additive has significant

effects on the performance of the solid-state solar cells. The performance of solid-state solar cells with the Li^+ ions is much higher than that of solar cells without Li^+, which is similar to previous studies.[6,7]

Time-of-flight secondary ion mass spectrometry (TOF-SIMS) depth profiles were also used to identify the element distribution in the sample with and without Li-TFSI and the results are shown in Supplementary Fig. 16 c and d. In a solar cell with Li-TFSI, Li ions occur in the sample after illumination, which has similar distribution of the Ti ions (Supplementary Fig. 16c). However, no Li ions are observed in the sample without Li-TFSI after illumination (Supplementary Fig. 16d). The results suggest that the inserted Li ions into TiO_2 come from the solid hole-transporting layer. Therefore, the Faradaic reaction also happens in a solid-state solar cell.

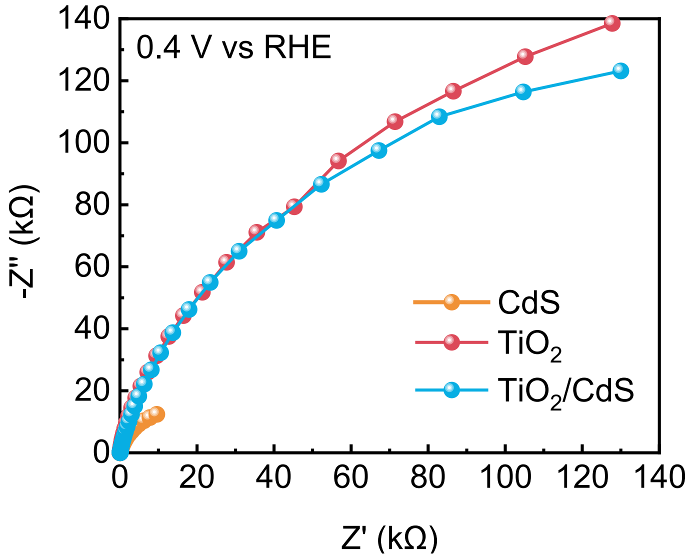

Supplementary Fig. 17| Electrochemical impedance spectroscopy of CdS, TiO_2 and TiO_2/CdS at 0.4 V_{RHE} in 1 M Na_2S aqueous solution in the dark.

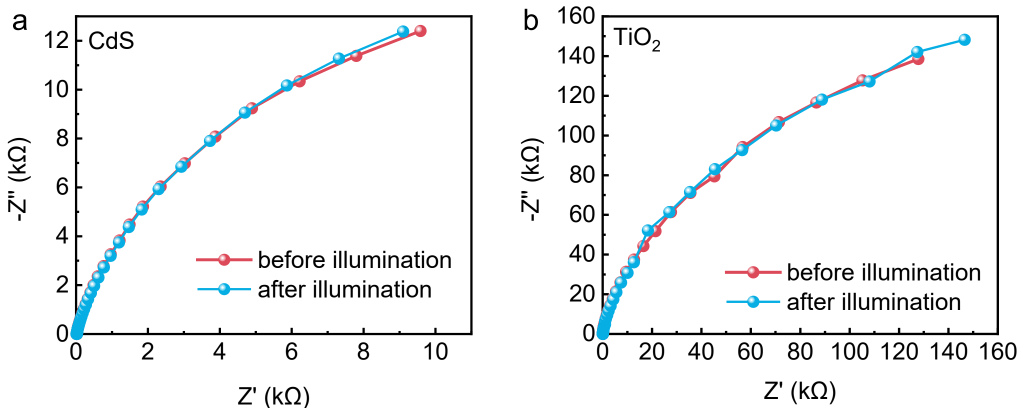

Supplementary Fig. 18| Electrochemical impedance spectroscopy of CdS (a) and TiO_2 (b) at 0.4 V_{RHE} in 1 M Na_2S aqueous solution before and after illumination; Light source: a full arc Xe lamp.

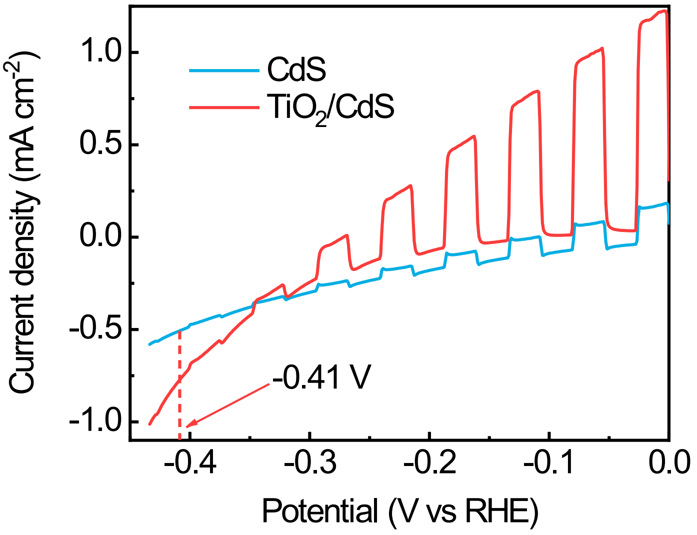

Supplementary Fig. 19| Linear sweep voltammetry curves of CdS in 1 M Na_2S aqueous solution under chopped full arc Xe lamp illumination.

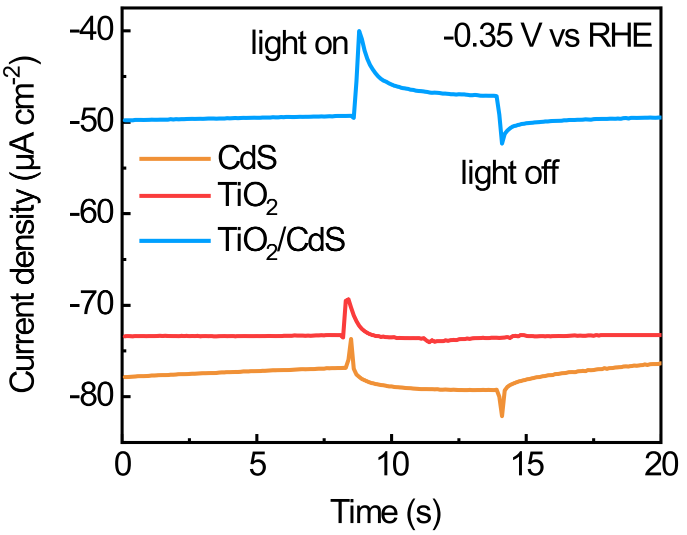

Supplementary Fig. 20| I-t curves of CdS, TiO_2 and TiO_2/CdS at -0.35 V_{RHE} in 1 M Na_2S aqueous solution; Light source: a Xe lamp with an AM 1.5 sunlight simulator filter, light intensity: 100 mW/cm^2.

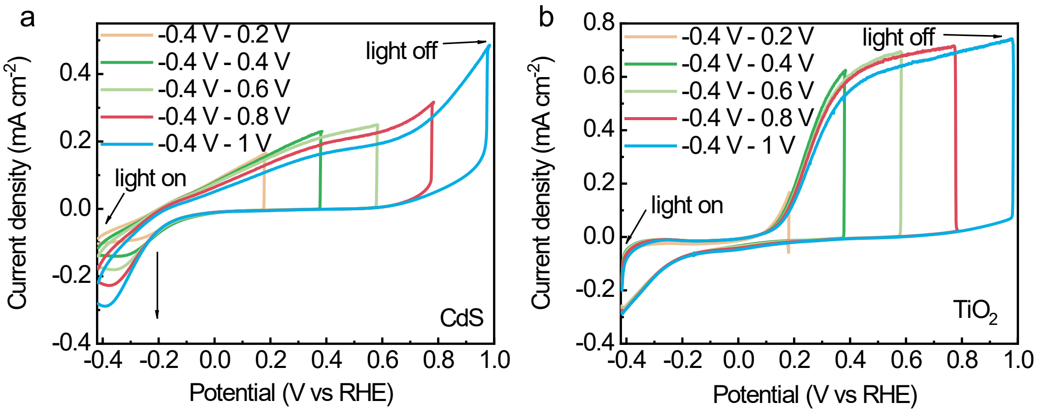

Supplementary Fig. 21| Cyclic voltammetry curves of CdS (a) and TiO_2 (b) in 1 M Na_2S aqueous solution; Light source: a Xe lamp with an AM 1.5 sunlight simulator filter; light intensity: 100 mW/cm^2.

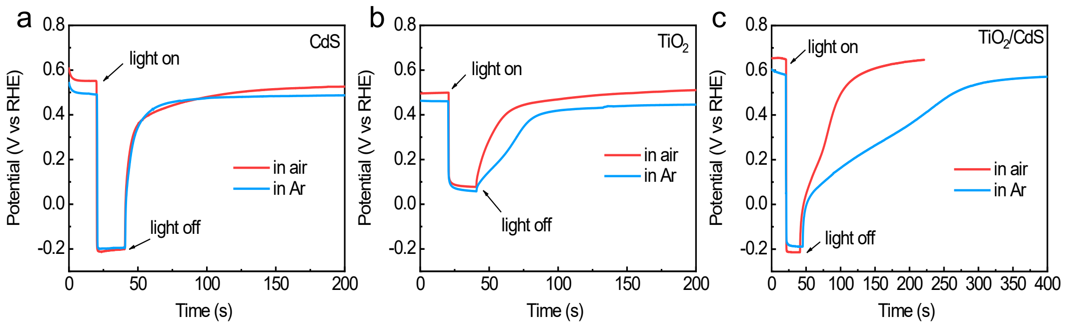

Supplementary Fig. 22| Open-circuit potential of CdS (a), TiO_2 (b) and TiO_2/CdS (c) in 1 M Na_2S aqueous solution with air and Ar in the dark and under illumination; Light source: a Xe lamp with an AM 1.5 sunlight simulator filter; light intensity: 100 mW/cm^2.

In order to investigate the open-circuit potential decay process more clearly, the open-circuit potential of CdS, TiO_2 and TiO_2/CdS were also measured in the electrolyte with air and Ar and the results are shown in Supplementary Fig. 22. The open-circuit potential decay of CdS is independent on the atmosphere (Supplementary Fig. 22a), which suggests that no Faradaic reaction happens in CdS under illumination. In contrast, the open-circuit potential decay of TiO_2 depends on the atmosphere and a much slower decay rate is observed on TiO_2 in the electrolyte with Ar than that in the electrolyte with air (Supplementary Fig. 22b). The results further confirm that the slow open-circuit potential decay of TiO_2 indeed comes from the re-oxidation of the reduced intrinsic Faradaic layer by oxygen. Therefore, the photo-reduced intrinsic Faradaic layer of $TiO_{2-x}(OH)_{2x}$ on the surface of TiO_2 can be re-oxidized by oxygen in the electrolyte under open circuit conditions.

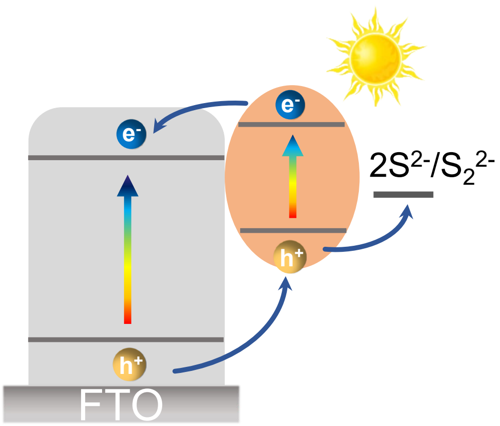

Supplementary Fig. 23| Interface charge transfer in a Type II heterojunction.

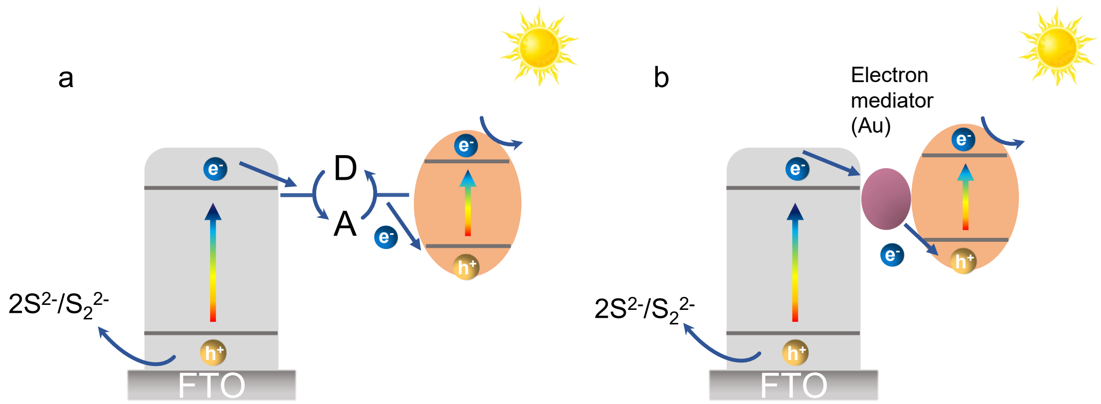

Supplementary Fig. 24| Interface charge transfer in an indirect Z-scheme heterojunction with redox shuttle (a) or Au (b) as electron mediator.

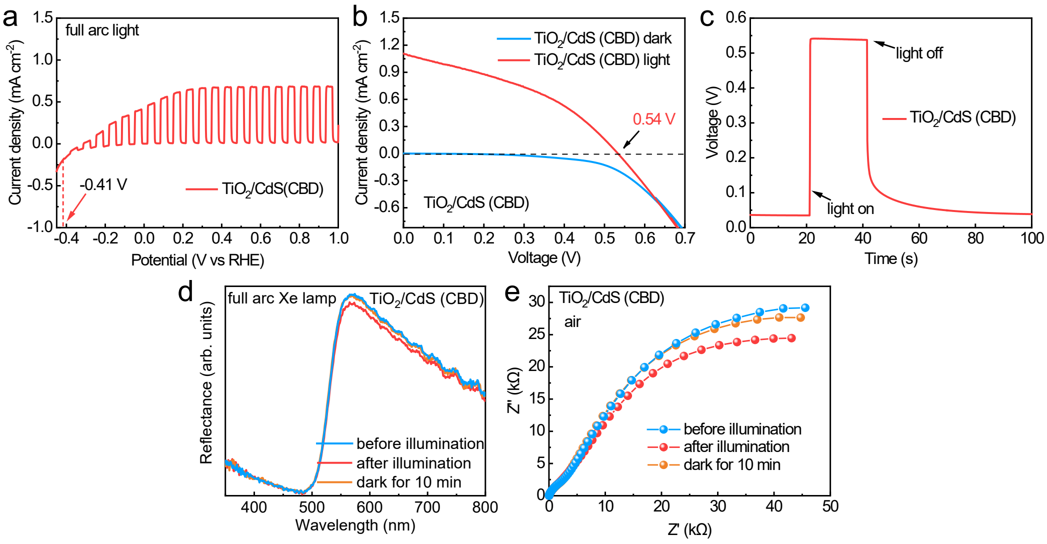

Supplementary Fig. 25| Linear sweep voltammetry curves of TiO_2/CdS (CBD) in 1 M Na_2S aqueous solution under chopped illumination (a); I-V curves (b) and V_{OC} (c) of TiO_2/CdS (CBD) quantum-dot sensitized solar cells in the dark and under illumination, electrolyte: 0.5 M Na_2S, 2 M S and 0.2 M KCl in water/methanol (3:7 by volume); Quasi in situ UV-vis reflectance spectra (d) of TiO_2/CdS (CBD) in 1 M Na_2S aqueous solution before and after illumination under full arc Xe lamp; Electrochemical impedance spectroscopy (e) @0.4 V_{RHE} of TiO_2/CdS (CBD) in 1 M Na_2S aqueous solution with air before and after illumination under full arc Xe lamp. Light source: a Xe lamp with an AM 1.5 sunlight simulator filter, light intensity: 100 mW/cm^2.

In order to investigate whether the Faradaic junction mechanism is related to the fabrication technology, we also coated CdS on the TiO_2 surface by chemical bath deposition (CBD) method. The preparation details are shown in Methods. The performance of TiO_2/CdS (CBD) was measured and the results are shown in Supplementary Fig. 25a-c. The TiO_2/CdS (CBD) indicates very close onset potentials and V_{OC} with the TiO_2/CdS (SILAR) sample. Moreover, the lower reflectance at the wavelength range of 520~800 nm (Supplementary Fig. 25d) and the decreased impedance semicircle (Supplementary Fig. 25e) after illumination are also observed in the TiO_2/CdS (CBD) sample, which are also similar to the TiO_2/CdS (SILAR) sample

(Figure 2c and 3e). These results suggest that the Faradaic junction mechanism is independent on the fabrication technology.

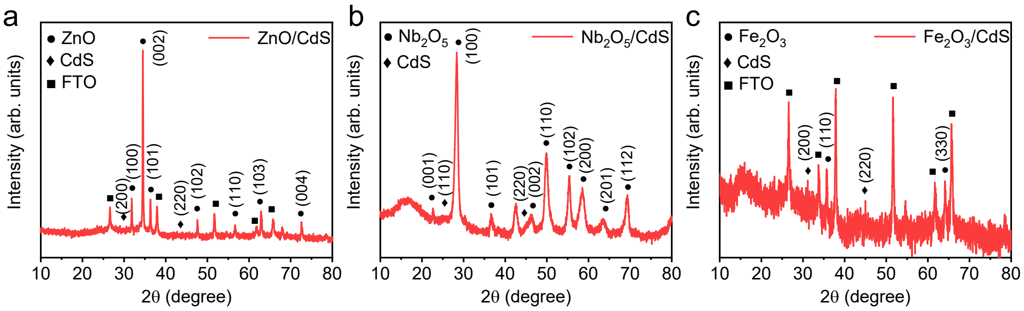

Supplementary Fig. 26| XRD patterns of as-deposited ZnO/CdS (a), Nb_2O_5/CdS (b) and Fe_2O_3/CdS (c) heterojunctions.

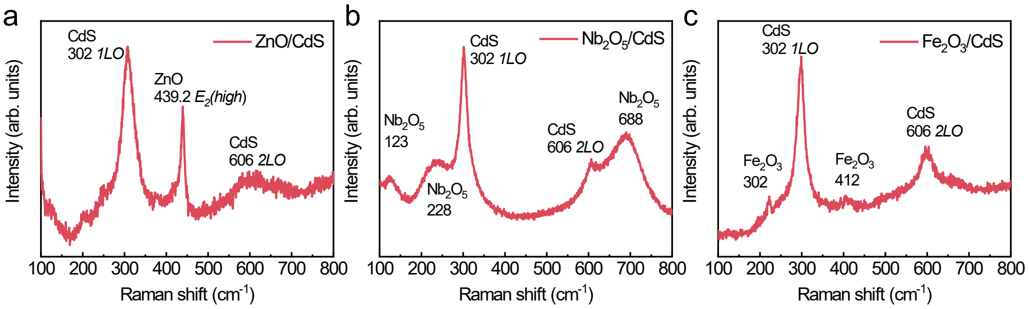

Supplementary Fig. 27| Raman spectra of as-deposited ZnO/CdS (a), Nb_2O_5/CdS (b) and Fe_2O_3/CdS (c) heterojunctions.

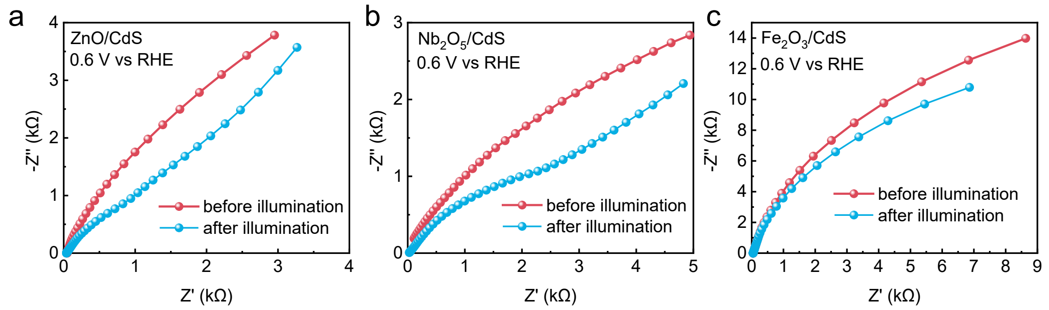

Supplementary Fig. 28| Electrochemical impedance spectroscopy @0.6 V_{RHE} of ZnO/CdS (a), Nb_2O_5/CdS (b) and Fe_2O_3/CdS (c) in 1 M Na_2S aqueous solution before and after illumination. Light source: a Xe lamp with an AM 1.5 sunlight simulator filter, light intensity: 100 mW/cm^2.

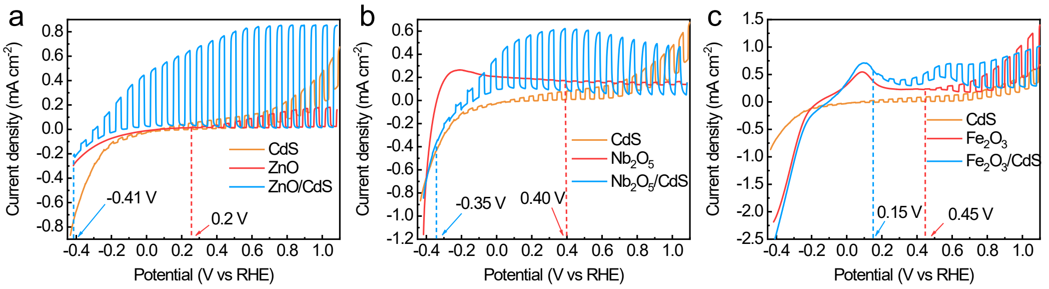

Supplementary Fig. 29| Linear sweep voltammetry curves of ZnO/CdS (a), Nb_2O_5/CdS (b) and Fe_2O_3/CdS (c) heterojunctions in 1 M Na_2S aqueous solution under chopped light illumination. Light source: a Xe lamp with an AM 1.5 sunlight simulator filter, light intensity: 100 mW/cm^2.

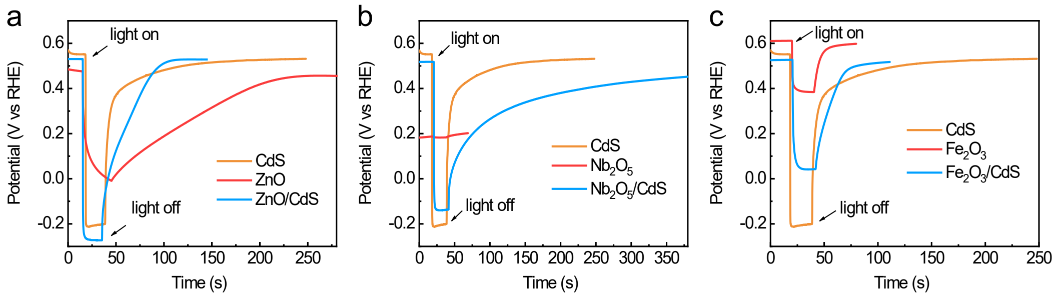

Supplementary Fig. 30| Open-circuit potentials of ZnO/CdS (a), Nb_2O_5/CdS (b) and Fe_2O_3/CdS (c) heterojunctions in 1 M Na_2S aqueous solution with air in the dark and under illumination. Light source: a Xe lamp with an AM 1.5 sunlight simulator filter, light intensity: 100 mW/cm^2.

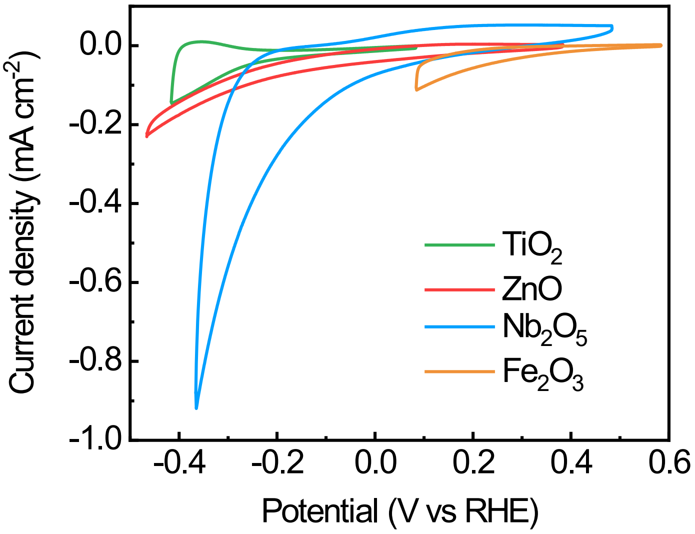

Supplementary Fig. 31| Faradaic potential windows of ZnO[8–11], Nb_2O_5[12] and Fe_2O_3[13,14] in 1 M Na_2S aqueous solution in the dark.

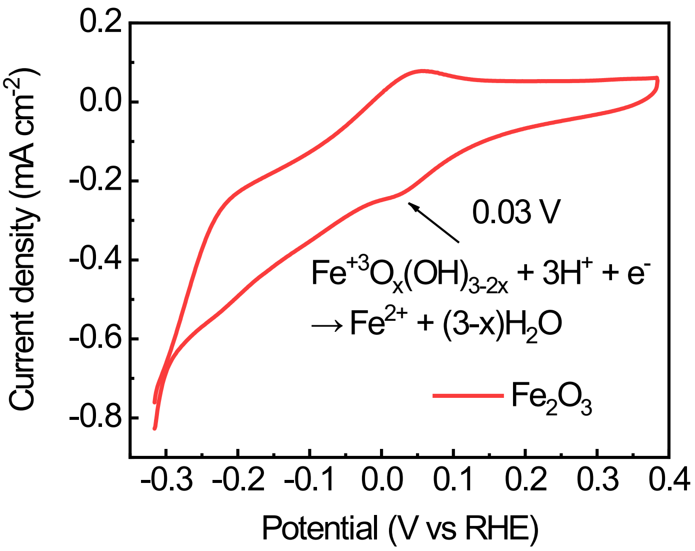

Supplementary Fig. 32| Cyclic voltammetry curves of Fe_2O_3 in 1 M Na_2S aqueous solution in the dark.

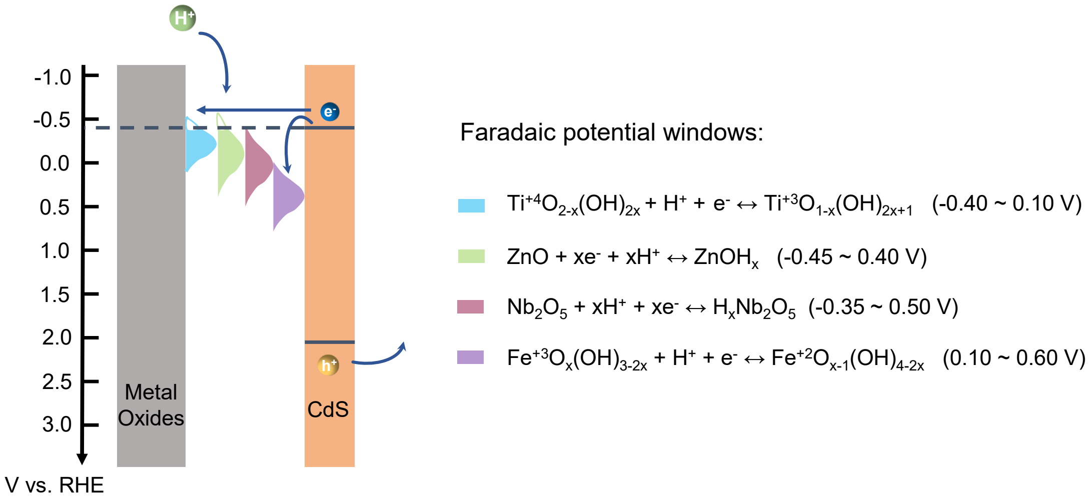

Supplementary Fig. 33| Interface charge transfer in TiO_2/CdS, ZnO/CdS, Nb_2O_5/CdS and Fe_2O_3/CdS heterojunctions under illumination.

References

1. Jing Wei-Xuan *et al.* Relationships between synthesizing parameters, morphology,

and contact angles of ZnO nanowire films. *Acta Phys. Sin.* **62**, 218102 (2013).

2. Yin, Z. *et al.* Mildly regulated intrinsic faradaic layer at the oxide/water interface for improved photoelectrochemical performance. *Chem. Sci.* **11**, 6297-6304 (2020).
3. Chen, W. *et al.* Efficient and stable large-area perovskite solar cells with inorganic charge extraction layers. *Science* **350**, 944-948 (2015).
4. Xiao, Y. *et al.* Band structure engineering and defect control of Ta_3N_5 for efficient photoelectrochemical water oxidation. *Nat. Catal.* **3**, 932-940 (2020).
5. Im, J.-H., Jang, I.-H., Pellet, N., Grätzel, M. & Park, N.-G. Growth of $CH_3NH_3PbI_3$ cuboids with controlled size for high-efficiency perovskite solar cells. *Nat. Nanotechnol.* **9**, 927-932 (2014).
6. Bach, U. *et al.* Solid-state dye-sensitized mesoporous TiO_2 solar cells with high photon-to-electron conversion efficiencies. *Nature* **395**, 583-585 (1998).
7. Murakoshi, K., Kogure, R., Wada, Y. & Yanagida, S. Solid State Dye-Sensitized TiO_2 Solar Cell with Polypyrrole as Hole Transport Layer. *Chem. Lett.* **26**, 471-472 (1997).
8. Schrauben, J. N. *et al.* Titanium and Zinc Oxide Nanoparticles Are Proton-Coupled Electron Transfer Agents. *Science* **336**, 1298-1301 (2012).
9. Chianella, C., Palombari, R. & Petricca, A. Electrochemical hydrogen doping of zinc oxide: A study of the oxide-proton conductor interface. *Electrochim. Acta* **52**, 369-372 (2006).
10. Hoyer, P. & Weller, H. Potential-Dependent Electron Injection in Nanoporous Colloidal ZnO Films. *J. Phys. Chem.* **99**, 14096-14100 (1995).

11. Braten, M. N., Gamelin, D. R. & Mayer, J. M. Reaction Dynamics of Proton-Coupled Electron Transfer from Reduced ZnO Nanocrystals. *ACS Nano* **9**, 10258-10267 (2015).

12. Gomes, M. A. B., Bulhões, L. O. de S., de Castro, S. C. & Damião, A. J. The Electrochromic Process at Nb_2O_5 Electrodes Prepared by Thermal Oxidation of Niobium. *J. Electrochem. Soc.* **137**, 3067-3070 (1990).

13. Cohen, M. & Hashimoto, K. The Cathodic Reduction of Gamma-FeOOH, Gamma-Fe_2O_3, and Oxide Films on Iron. *J. Electrochem. Soc.* **121**, 42 (1974).

14. Cox, A. & Lyon, S. B. An electrochemical study of the atmospheric corrosion of iron-II. Cathodic and anodic processes on uncorroded and pre-corroded iron. *Corros. Sci.* **36**, 1177-1192 (1994).
