# Supplementary material for: Faradaic junction and isoenergetic charge transfer mechanism on semiconductor/semiconductor interfaces
Source: Nat Commun. 2021 Nov 4;12:6363. doi: 10.1038/s41467-021-26661-6 (PMC8569189; doi:10.1038/s41467-021-26661-6)
Supplement: Supplementary file 2 — Lasing Reporting Summary [file 41467_2021_26661_MOESM2_ESM.pdf]

nature research

Corresponding author(s): Wenjun Luo

Lasing Reporting Summary

Nature Research wishes to improve the reproducibility of the work that we publish. This form is intended for publication with all accepted papers reporting claims of lasing and provides structure for consistency and transparency in reporting. Some list items might not apply to an individual manuscript, but all fields must be completed for clarity.

For further information on Nature Research policies, including our data availability policy, see Authors & Referees.

ü Experimental design

Please check: are the following details reported in the manuscript?

1. Threshold

Plots of device output power versus pump power over a wide range of values indicating a clear threshold

- [ ] Yes
- [x] No

In this study, a laser is only be used in a commercial Raman spectroscopy (spectuHoriba T64000, excitation wavelength ~ 488 nm) for sample characterization. Therefore, it is not necessary to provide the relevant parameters.

2. Linewidth narrowing

Plots of spectral power density for the emission at pump powers below, around, and above the lasing threshold, indicating a clear linewidth narrowing at threshold

- [ ] Yes
- [x] No

In this study, a laser is only be used in a commercial Raman spectroscopy (spectuHoriba T64000, excitation wavelength ~ 488 nm) for sample characterization. Therefore, it is not necessary to provide the relevant parameters.

Resolution of the spectrometer used to make spectral measurements

- [ ] Yes
- [x] No

In this study, a laser is only be used in a commercial Raman spectroscopy (spectuHoriba T64000, excitation wavelength ~ 488 nm) for sample characterization. Therefore, it is not necessary to provide the relevant parameters.

3. Coherent emission

Measurements of the coherence and/or polarization of the emission

- [ ] Yes
- [x] No

In this study, a laser is only be used in a commercial Raman spectroscopy (spectuHoriba T64000, excitation wavelength ~ 488 nm) for sample characterization. Therefore, it is not necessary to provide the relevant parameters.

4. Beam spatial profile

Image and/or measurement of the spatial shape and profile of the emission, showing a well-defined beam above threshold

- [ ] Yes
- [x] No

In this study, a laser is only be used in a commercial Raman spectroscopy (spectuHoriba T64000, excitation wavelength ~ 488 nm) for sample characterization. Therefore, it is not necessary to provide the relevant parameters.

5. Operating conditions

Description of the laser and pumping conditions
Continuous-wave, pulsed, temperature of operation

- [ ] Yes
- [x] No

In this study, a laser is only be used in a commercial Raman spectroscopy (spectuHoriba T64000, excitation wavelength ~ 488 nm) for sample characterization. Therefore, it is not necessary to provide the relevant parameters.

Threshold values provided as density values (e.g. W cm^{-2} or J cm^{-2}) taking into account the area of the device

- [ ] Yes
- [x] No

In this study, a laser is only be used in a commercial Raman spectroscopy (spectuHoriba T64000, excitation wavelength ~ 488 nm) for sample characterization. Therefore, it is not necessary to provide the relevant parameters.

6. Alternative explanations

Reasoning as to why alternative explanations have been ruled out as responsible for the emission characteristics
e.g. amplified spontaneous, directional scattering; modification of fluorescence spectrum by the cavity

- [ ] Yes
- [x] No

In this study, a laser is only be used in a commercial Raman spectroscopy (spectuHoriba T64000, excitation wavelength ~ 488 nm) for sample characterization. Therefore, it is not necessary to provide the relevant parameters.

7. Theoretical analysis

Theoretical analysis that ensures that the experimental values measured are realistic and reasonable
e.g. laser threshold, linewidth, cavity gain-loss, efficiency

- [ ] Yes
- [x] No

In this study, a laser is only be used in a commercial Raman spectroscopy (spectuHoriba T64000, excitation wavelength ~ 488 nm) for sample characterization. Therefore, it is not necessary to provide the relevant parameters.

8. Statistics

Number of devices fabricated and tested	☐ Yes<br>☒ No	In this study, a laser is only be used in a commercial Raman spectroscopy (spectuHoriba T64000, excitation wavelength ~ 488 nm) for sample characterization. Therefore, it is not necessary to provide the relevant parameters.
Statistical analysis of the device performance and lifetime (time to failure)	☐ Yes<br>☒ No	The Raman spectroscopy is measured by spectuHoriba T64000, excitation wavelength ~ 488 nm, which is a commercial and widely used instrument.
